# Improving measurement-invariance assessments: correcting entrenched testing deficiencies

**DOI:** 10.1186/s12874-016-0230-3

**Published:** 2016-10-06

**Authors:** Leslie A. Hayduk

**Affiliations:** Department of Sociology, University of Alberta, Edmonton, T6G 2H4 Canada

**Keywords:** Invariance, Factor analysis, Testing, Close fit, Structural equation model, SEM

## Abstract

**Background:**

Factor analysis historically focused on measurement while path analysis employed observed variables as though they were error-free. When factor- and path-analysis merged as structural equation modeling, factor analytic notions dominated measurement discussions – including assessments of measurement invariance across groups. The factor analytic tradition fostered disregard of model testing and consequently entrenched this deficiency in measurement invariance assessments.

**Discussion:**

Applying contemporary model testing requirements to the so-called *configural model* initiating invariance assessments will improve future assessments but a substantial backlog of deficient assessments remain to be overcome.

This article

- summarizes the issues,
- demonstrates the problem using a recent example,
- illustrates a superior model assessment strategy,
- and documents disciplinary entrenchment of inadequate testing as exemplified by the journal *Organizational Research Methods*.

**Summary:**

Employing the few methodologically and theoretically best, rather than precariously-multiple, indicators of latent variables increases the likelihood of achieving properly causally specified structural equation models capable of displaying measurement invariance. Just as evidence of invalidity trumps reliability, evidence of configural model misspecification trumps invariant estimates of misspecified coefficients.

**Electronic supplementary material:**

The online version of this article (doi:10.1186/s12874-016-0230-3) contains supplementary material, which is available to authorized users.

## Background

Structural equation models meld a measurement “model” composed of the causal connections between latent and observed variables, to a latent-level “model” composed of the causal connections between the latent-level variables. The measurement and latent model-components tended to be viewed as distinct because the measurement model-segment historically developed from the factor analytic tradition [1–3] while the latent-level model-segment followed the path analytic tradition [4–8]. These model-segments can be appropriately and beneficially statistically combined but the factor tradition of downplaying and evading model testing, conflicts with the path analytic tradition of attentive model testing. Researchers following factor analytic traditions and confronting failing models were inclined to report fit-indices rather than correct the problems detected through model testing [9].

Structural equation model measurement and latent-level specifications should be granted equal scrutiny because the latent level cannot function appropriately without proper measurement, and measurement is not assured unless the supposedly-measured latents function appropriately [10–12]. The question of *measurement invariance* arises when researchers consider whether an indicator item measures the same thing in different contexts – whether in different laboratories, different countries, different religions, at different times, or with different languages [13]. Comparing variables’ effects or means between groups makes sense when the same variables are being considered in the two groups. It is reasonable to compare attitudes/apples in one group with attitudes/apples in another group, but usually less instructive to compare one group’s attitudes/apples to another group’s desires/oranges. Comparing structural equation models demands attention to both the observed indicator variables and their underlying latent causes because even if the indicators are identical the underlying latent sources might differ between the groups. Asking a group of men how frequently they shave taps into something different than asking women the identical question.

Measurement-invariance assessments frequently begin with a factor-structured model that is progressively constrained by adding between-group equality constraints on the loadings, measurement error variances, and measurement intercepts. Changes in loadings (the causal actions leading from the latents to indicators) are usually granted priority because differences in loadings directly signal differences between the observed indicators and the underlying latents. If an indicator is more strongly responsive to a latent in one group than another, this signals a change in the causal source of the indicator – something different may be being measured in the two groups. Parallel comments apply to measurement error variances, intercepts, and other model coefficients [13]. Consequently, testing the tenability of between-group equality constraints on loadings, and other model coefficients, underpins assessments of measurement consistency or invariance. Assessing measurement invariance by investigating between-group coefficient constraints is reasonable as long as inattention to model testing has not already undermined the very foundation of this approach. Unfortunately, the concern for testing coefficient-constraints has not routinely extended to testing the baseline model containing the constrained coefficients [14].

A factor-structured model, called the configural model, often constitutes the initial model in the invariance testing process [13]. The configural model typically results from factor analyses which place each indicator under a specific factor. The clustering of indicators under latent factors is conveyed by requiring zero cross-loadings from each latent to the indicators of the other latent factors, and zero measurement error covariances (corresponding to the presumption of statistically independent errors). The identical clustering of indicators under latent factors in the two groups constitutes the basic configuration that grants the name *configural model*. The configural model places no between-group constraints on the estimated coefficients, so the loadings, measurement error variances, and other estimates may differ between the groups, though the clustering of indicators under latents (the placement of the loadings) retains the same configuration in both groups.

This raises the issue of whether or not the configural model, with its many zero loadings and other coefficients that are constrained to be identical in the two groups, must be carefully tested. The perspective taken here is that the configural model must be carefully tested [14] – which conflicts with the uncomfortably-common practice of treating significant inconsistency between the data and the configural model as “acceptable” or “tolerable” by appealing to fit indices such as RMSEA or CFI [13, 15-18]. Defending measurements as invariant on the basis of consistency between groups is untenable if the model’s structure is inconsistent with the world’s causal structure [19, 20]. If the configural model’s structure does not correspond to the world’s causal structure, asking about invariance between groups is asking whether the groups agree in their ***mis***representation of the connections between the indicators and the underlying latent variables! Measurement invariance makes sense not merely because the groups agree with one another but because there is between-group agreement *as well as consistency between the model and the worldly causal structures that provided the data*.

It is important to differentiate between model fit and model properness, because seriously causally misspecified/wrong factor models can fit. Hayduk [11], for example, illustrates that it is sometimes possible for a one-factor model to *perfectly fit* data generated by three real-world latent factors. It would be unreasonable to claim adequate or invariant measurement of one-factor if the real world contains three factors, not one! Given that causally wrong factor models can provide perfect-fit, it should be obvious that more extreme causal misspecifications can produce near-fit, or close-fit. The ability of importantly causally-incorrect models to nearly-fit makes it unreasonable to employ mere closeness-of-fit as the benchmark for assessing measurement, or measurement invariance. The appropriate measurement concern is not some indexed amount of ill fit or closeness to fit, but whether or not the data are consistent with the indicated number of underlying latents having the specified connections to the indicators [20]. Evidence of inconsistency between the model’s structure and the world’s structure destroys the very foundation of any claim that the model provides adequate measurement.

## Data and procedures

To illustrate both the problem and a helpful alternative, we consider an example discussed by Cheung and Lau [17] which used the publically available Work Orientations data for residents of the United States and Great Britain published in 1989 by the International Social Survey Program [21]. SPSS 22 [22] was used for calculation of the basic indicator statistics, and maximum likelihood estimates from covariance input were obtained via LISREL 9.1 [23]. The survey questions providing the indicators, their means, and standard deviations are provided in Table 1.

**Table 1:** The ISSP Work Indicators^a^

Indicator Wording	Designation here, and in Cheung & Lau	ISSP	Great Britain		United States	
			Mean	Std. Deviation	Mean	Std. Deviation
My job is secure	*y*_1_	V59	2.46	1.073	2.09	.977
My income is high	*y*_2_	V60	3.38	.956	3.22	1.009
My opportunities for advancement are high	*y*_3_	V61	3.29	1.030	3.00	1.115
My job has flexible working hours	*y*_4_	V67	3.16	1.218	2.82	1.192
My job is interesting	*y*_5_	V63	2.11	.852	2.12	.967
I can work independently	*y*_6_	V64	2.08	.838	2.09	.965
In my job I can help other people	*y*_7_	V65	2.28	.951	2.07	.913
{how often}…are you bored at work?	*y*_8_	V71	2.19	.937	2.24	.953
{how often}…do you have to do hard physical work?	*y*_9_	V69	3.46	1.253	3.48	1.186
{how often}…do you work in dangerous conditions?	*y*_10_	V72	4.09	1.115	3.97	1.164
{how often}…do you work in unhealthy conditions?	*y*_11_	V73	4.06	1.100	4.14	1.042
{how often}…do you work in physically unpleasant conditions?	*y*_12_	V74	4.16	1.067	4.10	1.022
Sex	*x*_1_	V85	1.44	.496	1.47	.499
Age	*x*_2_	V86	39.31	11.463	38.62	11.914

^a^ISSP = International Social Survey Program 1989. We refer to Great Britain (rather than C&L’s United Kingdom) because that is the designation used in the ISSP. N = 648 Great Britain, 823 United States. Items *y*_1_ to *y*_7_ had lead-in: “For each statement about your main job below, please circle one code to show how much you agree or disagree that it applies to your job. 1 = Strongly Agree, to 5 = Strongly Disagree, 8 = Can’t Choose.” Items *y*_8_ to *y*_12_ had lead-in: “Now some more questions about your working conditions. Please circle one code for each item below to show how often it applies to your work. 1 = Always, 2 = Often, 3 = Sometimes, 4 = Hardly Ever, 5 = Never, 8 = Can’t Choose”. *y*_8_ is reverse coded. Sex: 1 = male, 2 = female. Age: in years. Only those working 10 h per week or more for pay responded to the above questions. According to Cheung and Lau: *y*_1_ to *y*_4_ indicate quality of **Job Context**; *y*_5_ to *y*_8_ indicate quality of **Job Content**; and *y*_9_ to *y*_12_ indicate quality of **Work Environment**

## Analysis and context

**Fig. 1:**
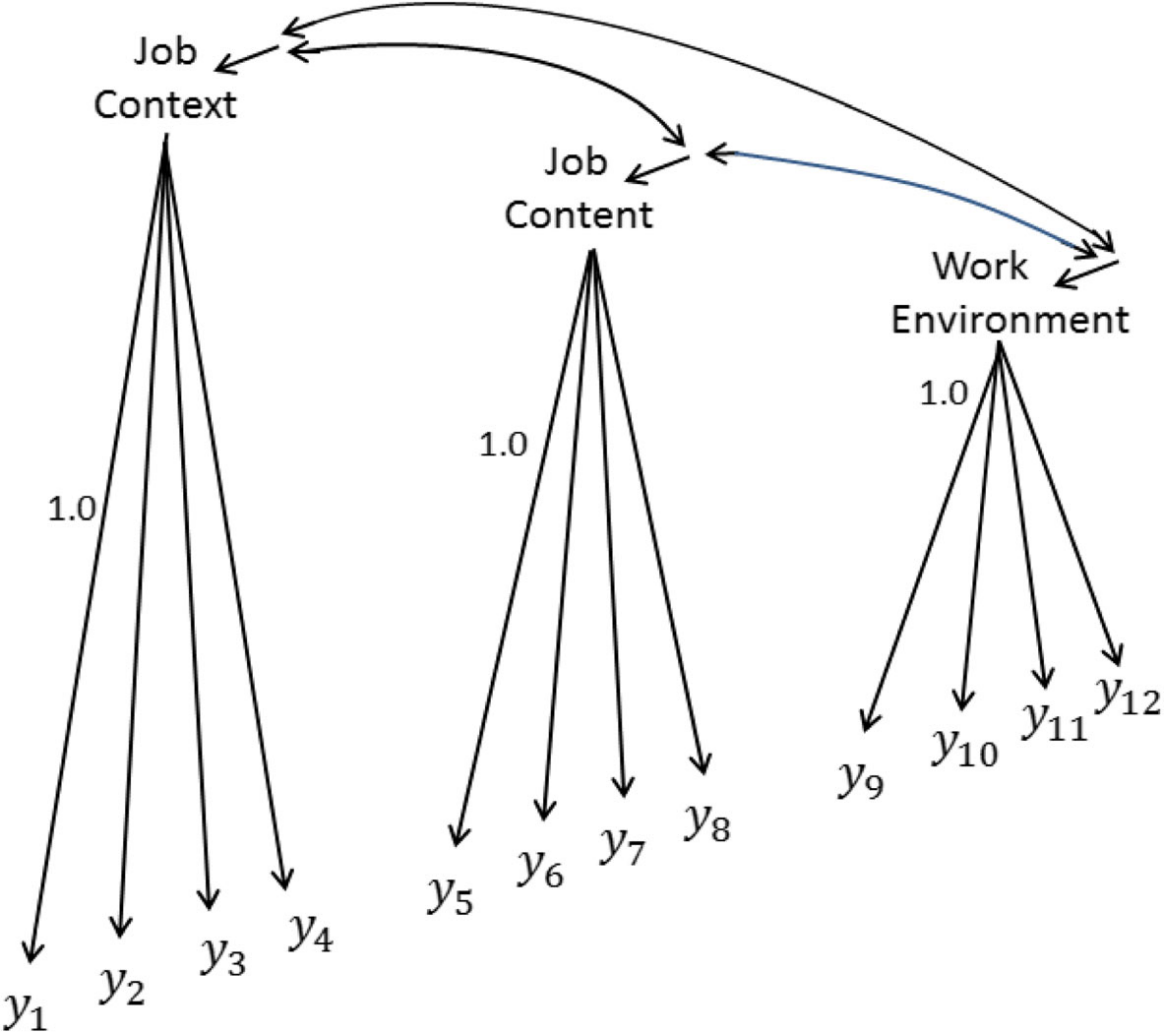
The Cheung and Lau (2012) configural model applies this model to each group

The configural model initiating the testing sequence investigated by Cheung and Lau [17] (hereafter C&L) is a factor model having four indicators of each of three latent variables: quality of *Job Conte* ***xt*** (*η*_1_), quality of *Job Conte* ***nt*** (*η*_2_), and quality of *Work Environment* (*η*_3_). This model (for one group) is depicted in Fig. 1. The configural model is the Fig. 1 model estimated simultaneously but separately for Great Britain (GB) and the United States (USA), with no additional constraints between the groups, so all the loadings and other model coefficients receive a unique estimate in each group. For clarity and replicability, we employed a 1.0 loading for the first indicator in each indicator-set to scale the corresponding latent variable. C&L focused on bootstrap procedures incorporating less-common scaling options, but these features of their analyses are tangential to our concern – which is the testing of the base or initial configural factor model.

C&L say: “As good model fit is a prerequisite for meaningful interpretation of BC bootstrap confidence intervals, it is necessary to ensure that the configural invariance model shows adequate model fit” [17]:172–173. C&L’s focus on Bias-Corrected bootstrapping is moot, and their concern for the adequacy of the configural model is laudable, but C&L’s statement nonetheless remains problematic. The problem centers on “adequate model fit”. C&L continued the effete factor tradition of disregarding evidence of measurement problems when they required that their configural model show only “acceptable model ***fit***” [17]:173 (emphasis added) rather than requiring consistency between the data and their configural model’s causal structure. Switching away from model-properness, to model-fit, is fundamentally problematic because it inappropriately pretends measurement could be reported as adequate and invariant even if their configural model’s causal structure was inconsistent with the world’s structure.

Unfortunately, C&L really meant, trusted, and depended-on, *fit* as opposed to respecting evidence of world-model causal inconsistency. C&L report that their configural model provides *χ* ^2^ = 399.6 with 102 degrees of freedom – namely with 51 degrees of freedom in each group. C&L did not report the corresponding probability, though anyone knowing that a *χ* ^2^ having many degrees of freedom is nearly normally distributed with mean equal to the degrees of freedom and variance twice the degrees of freedom (so a standard deviation is $\sqrt{2df}$) should not need a *χ* ^2^ calculator to determine C&L’s configural model’s *χ* ^2^ is about 20 standard deviations from the mean, and hence has a *p* < 0.000001.

That is convincing evidence of inconsistency between the data and C&L’s configural model. This probability informs us that there is essentially no chance that random sampling variations could account for the difference between the available data and C&L’s configural model, even after the configural model is supplemented with optimal loading and measurement error variance estimates in each group. To say this another way – there is essentially no chance that the observed data could have arisen via random sampling if the worldly causal forces were structured as in C&L’s configural model. And yet another way – something about C&L’s configural model’s specification must be changed in order to match or correspond to the world’s causal structure.

The model might be wrong for claiming three latents exist. If there were four or more latents in each country, it would be unreasonable to claim the indicators adequately measured three latents! Similarly, it would be unreasonable to claim adequate *invariant* measurement of three latents, if the available data are inconsistent with three modeled latent factors after constraining the loadings or other estimates to be *equal* between the GB and USA groups. In fact, all 24 models with between-group constraints reported in C&L’s Table 6 [17]:181 are highly significantly inconsistent with the International Social Survey Program data.

It is strange, but consistent with problematic factor analytic tradition, that C&L attend to significant increases in *χ* ^2^ upon insertion of additional between-group constraints (where about half the significant *χ* ^2^ changes are on the order of 10 to 20), yet they disregard the huge 399.6 *χ* ^2^ resulting from the constraints comprising their base configural model. They attend to comparatively small *χ* ^2^ changes resulting from estimating coefficients constrained to equality between the groups, but fail to attend to the huge *χ* ^2^ change resulting from the constraints providing the configural model which also required equality (in number of latents, loading placements, zero cross-loadings, and error covariances) between the groups.

One option for investigating what might be wrong with C&L’s configural model would be to check the modification indices for cross-loadings or error covariances in the configural factor model, but this approach is hampered by capitalization on chance, and it would fail to investigate the more difficult possibility that C&L’s configural model might contain the wrong number of latents. We chose the alternative approach of adding new evidence to the discussion by introducing some possible causes of the three latents C&L postulated. Given that men and women, and the young and old, likely have different work experiences and concerns, Sex and Age likely impact at least some of the work-focused indicators. If C&L’s three latents are in fact the appropriate causal foundations of the work indicators, Sex and Age should only impact those indicators indirectly through those three work latents. The covariances between Sex, Age and the work indicators provide new evidence regarding C&L’s postulated configural causal structure. If effects leading from Sex and Age to the three modeled work factors (now more accurately called work latents) are unable to account for the covariances between Sex, Age and the specified work indicators, that constitutes evidence suggesting the postulated work latent-factors are not the appropriate causal foundations of the indicators. This is an instance of single-indicated latents (Sex and Age) being used to more fully assess multiple-indicated latents/factors, namely C&L’s work latent-factors [24, 25].

We began by attempting to replicate the failure of C&L’s configural model with the International Social Survey Program data. The resultant *χ* ^2^ = 448.4 with 102 degrees of freedom and *p*=.0000, was similar though not identical to C&L’s *χ* ^2^. The difference in *χ* ^2^ values may be partially due to our analysis having three more respondents in each country than was reported by C&L. Cheung and Lau [17] report 645 and 820 cases, while we obtained 648 and 823 cases for Great Britain and the USA respectively. (Cheung & Rensvold [16] also report 823 USA cases in these data, so Cheung and Lau’s slightly reduced *N*s seem inexplicable). The difference in *χ* ^2^ values may also be partially due to our using maximum likelihood in LISREL rather than C&L’s bootstrapping in Mplus. Whatever the reason for the difference in *χ* ^2^ values, both agree C&L’s configural model is definitely inconsistent with the data. Furthermore, C&L’s configural model’s specification problems seem spread throughout the model rather than being localized – as evidenced by 9 and 11 (of the 24 possible) cross-loadings in the GB and US models having modification indices exceeding 4.0 (respectively).

**Fig. 2:**
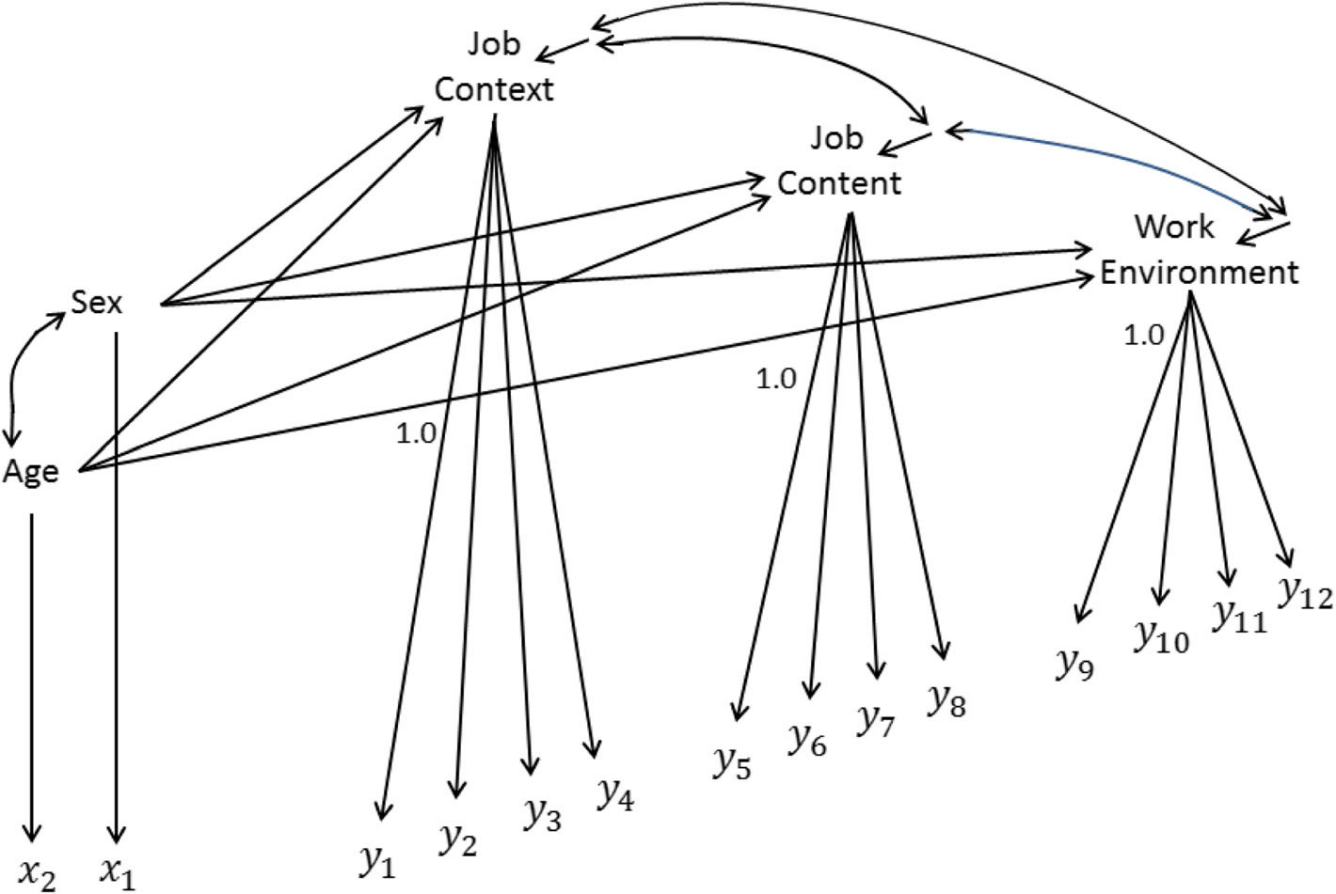
Sex and Age added to the Cheung and Lau (2012) configural model

Sex and Age were then added to the model as correlated single-indicated exogenous latent variables (each with 2 % measurement error variance) that were permitted to influence all three work latents (as in Fig. 2, for one group/country). (Single indicators of latents usually require fixed measurement error variances [24, 25] and the specified values elucidate and incorporate specific researcher assessments). No constraints were entered between the countries, so each country received its own estimates for both the new and original model coefficients. This specification ensures the work-measurement section of the model closely parallels C&L’s configural model, though the new model is a full structural equation model, and not just a factor-structured model. Sex and Age were selected because they were available in the International Social Survey Program data set, but any other appropriate causes or effects of the postulated latent factors could have been employed to check the adequacy of the factor structuring of C&L’s latents [24]: Chapter 1.

It is unsurprising that the Sex and Age supplemented model fails (*χ* ^2^ = 680.2 with 138 degrees of freedom and *p*=.0000) because this model’s structure cannot rectify the ill fit previously observed among the work indicators (where *χ* ^2^ was 448.4). (C&L’s three work latents’ loadings, variances and covariances continue to constitute the only explanation for relationships among the work indicators). Nonetheless, the substantial increase in *χ* ^2^ constitutes important news because this increase originates primarily in the work indicators’ covariances with Sex and Age. The 18 new degrees of freedom in each group come from attempting to explain 24 new covariances (between Sex, Age, and the 12 work indicators), with the six new effects leading from the Sex and Age latents to the three work latent “factors”. The substantial jump in *χ* ^2^ signals that the six latent effects of Sex and Age on the work-latents are insufficient to account for the 24 covariances between Sex, Age and the work-indicators. The standardized covariance residuals for 10 and 13 of these 24 covariances exceed 2.0 in GB and the US respectively, and hence would be significant if tested individually. If C&L’s configural model’s work latents constituted the correct causal foundations of the work indicators, those three latents would be able to appropriately distribute the causal impacts of Sex and Age to the work indicators. The multiple inconsistencies between the observed Sex and Age covariances and the covariances required to conform to C&L’s configural work-latent work-indicator specification, clearly report that the work latents in C&L’s configural model are inconsistent with the new evidence.

Another perspective on this is obtained by specifying Sex and Age in an all-η model (so the Sex and Age latents become *η*_4_ and *η*_5_) [26]. This produces the same estimates and ill fit of the work-indicators’ covariances with Sex and Age as reported for the Fig. 2 model above, but provides additional modification indices corresponding to potential effects leading directly from Sex and Age to each work indicator. Of the 24 potential “loadings” leading from the Sex and Age latents to the work indicators, 10 and 13 of the modification indices exceed 4.0 for GB and the US respectively. (As in the basic C&L configural model, with no Sex or Age, many of the cross-loadings from the work-latents to the work-indicators continued to have modification indices exceeding 4.0). Each real direct Sex or Age effect to a specific work indicator would challenge C&L’s presumption that three work latent-factors constitute the causal foundations of the work indicators. Such effects are inconsistent with their configural model’s causal structure because each direct effect to a work-indicator makes Sex or Age an additional latent common-cause of the work indicators – via that direct effect and the indirect effects of Sex and Age functioning through the work-latents. Thus both the covariance inconsistencies reported by the jump in *χ* ^2^, and the modification indices potentially connecting Sex and Age to specific work indicators, report that the work latent “factors” in C&L’s configural model are problematic, and that more, and/or different, latents actually produce the work-indicators.

We caution that we are NOT recommending following the modification indices to obtain fit, because problems created by an incorrect number of underlying latent factors cannot be resolved by following the modification indices. Indeed, the weakness of modification-index coefficient suggestions becomes evident if you notice that modification indices suggesting effects leading directly from Sex or Age to the work indicators do not correspond to the coefficients suggested by the modification indices for the cross-loadings or work-indicator error covariance in the basic C&L model. That is, following the original C&L model’s modification indices would not have addressed the kinds of model misspecifications being currently encountered. Similarly the current modification indices might improve fit without correcting the underlying problems – which may require changing the number and identity of the underlying latent variables being measured by the available work indicators.

So both the previously available evidence from within the set of work indicators themselves (as reported by C&L), and the new evidence from attempting to connect the work indicators to Sex and Age as exogenous causes, speak against the causal structuring of the work-latents in C&L’s configural model. But there remains the possibility that only the work indicators surviving C&L’s additional invariance investigations might fare better than the full set of indicators. C&L’s subsequent investigation of invariant intercepts might have coincidentally weeded out some covariance-problematic indicators, leaving only indicators appropriately modeled by C&L’s work latents.

This was investigated by setting up a model similar to Fig. 2, but employing only the work indicators C&L report as additionally displaying intercept, or scalar, invariance. C&L report the relevant indicators: for *Work Content* as *y*_5_, *y*_6_, and *y*_8_; for *Work Environment* as *y*_9_, *y*_10_, and *y*_12_; but left *Work Context* to be indicated either by the pair *y*_1_ *y*_4_ or the pair *y*_2_ *y*_3_ “based on theoretical interpretation and the research question” [17]:178. Using the *y*_2_ *y*_3_ pair for *Work Context*; *y*_5_, *y*_6_, *y*_8_ for *Work Content*; and *y*_9_ *y*_10_ *y*_12_ for *Work Environment*, along with Sex and Age, results in a model that continues to be severely inconsistent with the covariance data (*χ* ^2^ = 287.0 with 54 degrees of freedom and *p*=.0000) and displays the same general pattern of inconsistencies reported above. Using the *y*_1_ *y*_4_ pair produces similar disconfirmation but with additional evidence that *y*_1_ *y*_4_ in the GB group are too inconsistent with one another to support any reasonable covariances among the work latents. Collectively, these observations convincingly report that appealing to intercept-consistency cannot dispel, or overcome, the covariance-inconsistencies introduced by overlooked configural model causal misspecification.

We estimated one additional model employing Sex, Age, and two indicators of each work latent: *y*_2_ and *y*_3_ for *Work Content*, *y*_5_ and *y*_6_ for *Work Context*, and *y*_9_ and *y*_10_ for *Work Environment*. This model also fails to match the covariance data (*χ* ^2^ = 132.9 with 12 degrees of freedom and *p*=.0000) and displays the same scattered pattern of residual ill covariance fit, and numerous substantial modification indices connecting Sex, Age, and the work latents (via cross-loadings) to the work indicators. This instructs us that even pairs of indicators can sometimes detect problematic configural models, and implicitly instructs us that appropriate causal model specifications for these particular data may require some single-indicated work latents [25]. The diagnostics in the paired-indicators model became more focused than the diagnostics for the models having multiple indicators, and highlighted specific theoretical/methodological issues and options. This suggests it may be useful to begin measurement invariance testing with a configural model containing the few best indicators, rather than beginning with multiple indicators.

## Summary and discussion

Cheung and Lau [17] are not alone in disrespecting evidence of misspecification of the configural model initiating measurement invariance assessments. Indeed, there is a long and inglorious history of disrespect of configural model testing among even oft-cited foundational papers. Byrne, Shavelson and Muthen, for example, say that “A nonsignifiant *χ* ^2^ (or a reasonable fit as indicated by some alternate index) is justification that the baseline models fit the observed data” [15]:457. Notice the problematic focus on *fit* rather than on whether or not there is evidence the configural model is improperly causally structured. Byrne, Shavelson and Muthen’s configural factor models had *χ* ^2^ values more than a dozen standard deviations from the mean (and hence *p* values < 0.000001), and even after 5 modification-index prompted changes both groups remained significantly inconsistent with the data – one with so small a *p* value that it could only be reported as 0 by two different web *χ* ^2^ calculators. These models’ inconsistency with the data, directly contradicts Byrne, Shavelson and Muthen’s claim that their baseline configural model constitutes a “reasonable representation of the data” [15]:460.

A decade later, Vandenberg and Lance [13] said: “Overall model fit refers to evaluating the ability of the a priori model to (at least approximately) reproduce the observed covariance matrix” [13]:43), where the laxity of “at least approximately” is obvious, and where the concern is again inappropriately expressed as *fit* rather than measurement’s requirement of proper model causal specification. And nearly yet another decade later Schmitt and Kuljanin [27] reported that a configural model with *χ* ^2^ = 1183.86 and df = 174 whose *p* is reported as < 0.01, but that is also < 0.000001, “was accepted because considerable prior research confirmed the discriminant and convergent validity of these items” [27]:218 – as if clear evidence of problems in the current model’s specification could be justifiably disregarded because it would conflict with others’ claims! Schmitt and Kuljanin acknowledge that their review of more than 80 recent measurement invariance studies discovered that what authors “accepted as adequate evidence of configural invariance varied considerably across studies” and that what “constituted adequate fit was invariably subjective” [27]:212 – again notice the misguided emphasis on *fit*, which easily but inappropriately translates into *fit*-indices rather than concern for testing the causal properness of the model.

In that same year Meade, Johnson and Braddy [28] provided a statistically sophisticated simulation of configural measurement invariance testing, which unfortunately failed to acknowledge the study’s key limitation, namely that it disregarded the power of tests to differentiate between factor-structured configural models and non-factor worldly-models that can be confused with factor structures [11]. By simulating minor and intentionally-trivial factor model variations, while disregarding the more challenging issue of detecting incorrect non-factor structures, Meade et al. contributed (possibly unintentionally) to the myth that *N*-based power only detects trivial problems. Understanding that important model misspecifications can mimic the minor covariance residuals resulting from trivial factor-model misspecifications [11] makes it a glaring statistical mistake to claim that only trivial things become detectable with increasing *N*. Subsequently, downgrading *χ* ^2^ on the basis that it is “highly sensitive to sample size” [28] becomes a backhanded way of slighting the power provided by large samples – power capable of potentially detecting important model misspecifications. The supposed excuse of *χ* ^2^ being highly sensitive to sample size becomes a demonstration of factor-model myopia (seeing only factor-structured alternatives and misspecifications) and not a reasonable scientific response to a world whose causal features are currently unknown, and potentially not factor structured [29]. Fit-index-propelled disregard of evidence of model causal misspecification has undoubtedly led to more than a few optimistic-yet-erroneous measurement invariance reports.

Several observations seem warranted. First, if a researcher intends to investigate the invariance of measurements between groups, the basic model structure – the configural model initiating the invariance assessment – must be consistent with both groups’ data. Evidence of inconsistency between the configural model and either group’s data, may be signaling that the model contains incorrect latents. Incorrect latents render all “measurements”, including “measurements” that are consistent between groups, dubious because measurement is meaningless if the modeled latents do not correspond to worldly features [20]. When assessing measurement, evidence of invalidity trumps reliability [30].

Second, configural models initiating invariance testing need not be factor structured. It is reasonable to start with a full structural equation model that includes exogenous variables like Sex and Age. Indeed, it seems preferable to begin with a configural model whose latent structure is consistent with the researchers’ theory, their methodological understanding, and the data. Measurement and measurement-invariance assessments should be integrated with latent level structural understandings. Latents are known through their indicators – the basic factor claim – but latents are *not only*-known through their indicators. Latents are *also* known through the latent-level causal structures in which they participate – like Sex and Age structures [10, 31]. A substantial but avoidable factor-bias, and corresponding latent-theory weakness, accompanies routinely initiating measurement invariance testing with factor-structured configural models.

Third, it seems self-destructive to begin invariance testing with multiple indicators of factor-latents whenever it is likely to be difficult to obtain reasonably-functioning indicators. Occasionally, obtaining adequate measurement with even pairs of indicators may prove difficult – recall the failure of C&L’s model with only two indictors per work-latent. If a factor-structured configural model fails, and retaining the full set of indicators is desired, add latents instead of persisting with problematic factor structuring of the indicators. Again, validity trumps reliability when assessing measurement.

Fourth, researchers are urged to think causally about *all* their modeled variables. The covariances within each set of indicators, the covariances between diverse sets of indicators, and the covariances between latents, all result from productive/impactful/consequential effects in the real world. Faithful modeling of the worldly causal structures is required to attain adequate and invariant measurements. If the model’s structure is inconsistent with the indicators’ worldly causal milieu, the very notion that the indicators are *measures* is rendered dubious – even if those indicators function invariantly (consistently incorrectly). Yes, even in the context of invariance, validity concerns trump reliability when assessing measurement. Our configural models must structurally mirror worldly causal impacts if they are to testify to the adequacy and invariance of measures of worldly features [19, 20, 32].

The causal considerations should include the possibility of context-dependent causal impacts. For example, the above models represented Sex as having effects on C&L’s work latent variables, whereas proper measurement might require modeling statistical interactions because the relevant latent causal effects may differ between the sexes. For example, physically demanding work and unhealthy work conditions might be embedded in different causal networks for males than females, due to differences between typical male and female work environments. If so, the configural model should contain interactions with Sex when assessing between-country invariance. In general, understanding a latent factor as “something common to the items” is likely to be too causally imprecise to support a meticulously-causal configural model. For example, causal consideration of C&L’s factor-structured models requires considering whether the ***same*** basic latent variable Job Content *causes* workers to feel both that their job helps other people *and* that their job is interesting; and to consider whether the latent Work Environment *causes* both doing hard physical work and unhealthy work conditions (like exposure to diseases or dangerous chemicals). Our focus on statistical matters means that we, like C&L, are limited in the depth to which we can investigate the work indicators’ methodology and causal embeddedness, but the important point remains that proper or valid causal specification, however complex, constitutes a mandatory foundational requirement for measurement invariance assessments.

## Disciplinary-entrenchment of problems

The invariance testing problem illustrated above is more strongly entrenched in, and will be more difficult to dislodge from, some disciplines than others – presumably the disciplines committed to factor analysis. Researchers in such disciplines might consider the archived back-story to the current publication, namely SEMNET exchanges between Gordon Cheung and Les Hayduk in April, 2015 [33]. Those discussions resulted in an earlier version of this article being submitted to *Organization Research Methods.* Cheung is a senior scholar who is on the editorial board of *Organizational Research Methods,* and Cheung and Lau’s [17] article appeared in *Organization Research Methods,* so it seemed appropriate for that journal to participate in correcting a problem it helped (possibly unknowingly) propagate. The manuscript was rejected, with no invitation for resubmission or response to the reviews, by the editor (James LeBreton) in agreement with the associate editor’s (Adam Meade’s) recommendation – which pointed out that “Fundamentally, the reviewers do not agree with the philosophy espoused in the manuscript related to the necessity for sole reliance on chi-square as a method for testing”. It is patently silly to disregard the “sole” strongest-available testing on the basis of an alternative philosophy espousing weaker/deficient testing, so we might be inclined to laugh off this comment as reflecting the philosophical folly of junior scholars unfamiliar with the recent literature [10–12]. But the editor later indicated the reviewers “are individuals who are currently serving on the editorial boards of a number of leading journals in psychology, management, and quantitative methods. All three reviewers have served as chief editor or AE at one (or more) of the leading journals in their fields”. The appropriate academic response to this selectively-entrenched testing deficiency is to air the disagreements. To that end, I provided an Additional file 1 containing the original *ORM* manuscript, some editorial correspondence, and the anonymous reviews into which I inserted responses providing the contrasting testing philosophy. (Both editors of *Organizational Research Methods*, and their publisher, have agreed to the publication of their editorial correspondence and the peer review reports under Creative Common license). I invite you to weigh each spat, render your adjudication, and employ the victorious arguments to improve invariance testing. If the journals in your area do not yet exhibit a “philosophy” of respecting *EVIDENCE* of model misspecification and invalidity, you will have to either submit to a philosophy of evidence-disrespect, or become an agent of change [34].

## Conclusion

Ensure that a theory-appropriate and methods-appropriate causal configural model is consistent with your indicators before moving to any other steps in measurement invariance assessment.

## Additional file

Additional file 1: Documenting Entrenched Testing Deficiencies. (PDF 934 kb)
