# Supplementary material for: Improving measurement-invariance assessments: correcting entrenched testing deficiencies
Source: BMC Med Res Methodol. 2016 Oct 6;16:130. doi: 10.1186/s12874-016-0230-3 (PMC5052924; doi:10.1186/s12874-016-0230-3)
Supplement: Additional file 1: — Documenting Entrenched Testing Deficiencies. (PDF 934 kb) [file 12874_2016_230_MOESM1_ESM.pdf]

This **Additional File** documents entrenchment of problematic invariance-testing practices in *Organizational Research Methods* via the reviewers' and editors' responses to an earlier version of this article submitted to that journal. This file contains: a) the editor's and associate editor's rejection letters, b) the reviewers' comments – and inserted responses/replies to those comments, c) the manuscript submitted to *Organizational Research Methods*, and d) a post-review exchange of emails (inserted at the editor's request).

==

Organizational Research Methods - Decision on Manuscript ID ORM-15-0075

james.lebreton@psu.edu **via** **manuscriptcentral.com**

to me, awmeade

23-Jun-2015

Dear Dr.Hayduk,

Please see the letter below from the associate editor (AE), Dr. Adam Meade, regarding the status of your manuscript titled, "Improving Measurement-Invariance Assessments: Correcting Entrenched Deficiencies in Testing," submitted for review and publication consideration to Organizational Research Methods (ORM). The reviewers' comments follow immediately after Dr. Meade's letter.

In summary, after carefully reading your manuscript in combination with the reviewers' collective comments, Dr. Meade is recommending that this version of your manuscript not be accepted for publication in ORM and that ORM not extend an invitation to revise and resubmit your manuscript for one more review. I fully concur with his recommendation. Please consult Dr. Meade's letter and the reviewers' comments as to the details and reasoning underlying this decision. As authors ourselves, we know how disappointing this decision is to you. It is our sincerest hope, however, that the comments provided below will prove useful to you as you move forward with the manuscript.

Despite this decision, we hope that you will continue to consider ORM as a viable outlet for any future methodologically and/or statistically oriented manuscripts. Thank you for submitting your manuscript to Organizational Research Methods.

Sincerely,

James M. LeBreton, Editor

Organizational Research Methods

Sage Publications, Inc. (http://orm.sagepub.com/)
Sponsored by the Research Methods Division, Academy of Management (http://division.aomonline.org/rm/)
Housed in the Department of Psychology, Pennsylvania State University (http://psych.la.psu.edu/)

Associate Editor's Letter to the Author:

Associate Editor: Meade, Adam
Comments to the Author:
I have received three reviews of Manuscript ID ORM-15-0075 entitled "Improving Measurement-Invariance Assessments: Correcting Entrenched Deficiencies in Testing" which you submitted to Organizational Research Methods (ORM). The reviewers have a strong record of publication and have provided detailed and thoughtful reviews. Their reviews are included at the end of this letter (and an attached file). I also independently read the paper prior to reading the reviews.

The reviewers noted a number of positive features of your manuscript, but they also identified several serious limitations. In general, they were not particularly positive about the manuscript nor the possibility for revision. Therefore, based on the comments provided by the reviewers and my own assessment of your manuscript, I regret to say that I will not be able to recommend that your work be published nor that you be invited to revise and resubmit the manuscript.

In spite of this disappointing news, let me begin by saying that I believe there are a number of promising aspects to your paper. In particular, the notion of conducting invariance tests within the broader SEM framework is intriguing.

The reviewers offer many excellent comments on ways that you might consider altering your current manuscript and I will not repeat all of them here. Fundamentally, the reviewers do not agree with the philosophy espoused in the manuscript related to the necessity for sole reliance on chi-square as a method for testing (reviewer 1, comment 5; reviewer 2, comment 1; reviewer 3, comment 1). I believe much of this philosophical disagreement relates to the distinction between significance testing and effect sizes. While this paper is founded in invariance testing, much of these arguments relate to the configural model which is, in effect, just the fit to of the measurement model in two groups simultaneously. The nature of the debate relates largely to the strict assumptions placed on the baseline model. Most psychometricians would consider cross-loadings that are non-zero but very small (e.g., .05) to be practically meaningless. The chi-square statistic is not an effect size and with very large samples, it will reach statistical significance in presence of small but non-zero cross-loadings which have no meaningful or important substantive interpretation. Given the imprecision inherent in psychological and organizational measurement, most researchers consider the requirement of absolute zero cross-loadings to be excessively stringent. The solution you advocate is to eliminate indicators (which of course will eliminate the requirement of exact zero cross-loadings). As reviewer 3 (comment 4) notes, the effect of this is to reduce reliability of scales and is generally poor psychometric practice. It may also likely change the meaning of the construct. For instance, a very broad construct (such as any of the Big 5 personality traits) would likely be construct deficient with few indicators.

As an author, I know the enormous amount of work that goes into a study such as this one and I understand the disappointment of this letter. However, I sincerely wish you the best of luck in your work and hope that you can find a suitable outlet for your manuscript. I also I thank you for submitting your work to ORM and hope you will continue to do so in the future.
Kind Regards,

Adam W. Meade
Associate Editor, Organizational Research Methods

(Reviewers' comments with my/Les-Hayduk's replies to their comments inserted in bold italics.)

Reviewer(s)' Comments to Author:
Reviewer: 1

Comments on "Improving measurement-invariance assessments"
This paper addresses an important issue and is well written. However, I am not sure it represents a significant contribution. The following are my comments and questions.

1. Most important for me is the question of how one knows the "correct" model before data have been collected. Without knowledge of the correct model, a solution to this problem is not obvious to me.

We never "know" we have "the correct model" before or after data collection (that is a reason for testing the configural model), so this reviewer may never encounter an obvious "solution".

2. On page 2, you state that the configural model most often results from exploratory factor analysis. I think it may be more frequent that investigators start with a theory of the nature of the construct and which indicators represent each factor. Items are usually written to reflect specific constructs.

I expect that the factors to be represented with specific items often had exploratory backgrounds, but I deleted the term exploratory to remove this distraction.

3. Perhaps a minor issue in this context, but you use the first item in each factor to set the scale for that factor. There is a small body of literature indicating that care should be taken to select an indicator that is itself invariant to scale the factor.

Agreed, but given that the testing fails to locate any specific invariant C&L's items, the option of selecting an invariant item was not available.

4. On the bottom of page 3 and top of page 4, you state that the important problem is that the data are consistent with the specified number of underlying latent factors and their modeled connections to the indicators. That may be right but how does one know a priori what those connections might be.

Again, we do not "know" – that is why we test, and respect evidence that our current knowledge is deficient.

I agree that care should be taken in the construction and writing of indicators, but this will start ensure knowledge of the factor structure.

Yes construct indicators carefully, but do NOT DISREGARD EVIDENCE that a model's factor structuring is problematic. Be open to a wider range of latent-indicator specification possibilities.

5. Is not chi-square a function of N so that if you collect enough data even very small departures from the configural model will yield large and statistically significant values of chi-square?

Increasing N increases the power to detect only some, not all, problematic models. And the seriousness or importance of the misspecifications in models does NOT trustably coordinate with the amount of model ill fit (Hayduk 2014a). It is shameful to disregard significant model ill fit when even slight ill fit can be reporting serious model misspecification (Hayduk, 2014b).

Perhaps you should focus on practically significant departures from the configural model. It might be difficult to provide a practical outcome, but reliance on a statistical significance test alone does not seem the way to go to me.

It is a statistical mistake to believe that the "practical significance" of model misspecification is told to us by the amount of ill-fit (Hayduk, 2014a,b). Addressing and correcting model misspecification requires attending to the strongest available evidence – evidence that is currently provided by the chi-square test. If a stronger test becomes available, use it. Appealing to a weaker test, or displacing strong testing by switching to fit indices is statistically deficient because it constitutes disregarding evidence that may be signaling important model misspecification.

6. Can you use any two variables in place of sex and age to make the same point? This doesn't seem reasonable.

Any variables appropriately modeled as causes or effects can be used to test the configural structure (Hayduk, 1996 Chapter 1). A statement to this effect has been added to the text.

7. How does one know which variables will be good indicators? I assume this will be the result of some empirical search for the best. How will this affect the identification of the true latent factors especially in the case of small sample sized and a large number of indicators?

Invariance assessments are only possible when sample size is sufficient to support the configural model. If the researcher has sufficient sample size to engage in invariance assessment, they have sufficient sample size to attend to the testing of their basic configural model. Notice that the latents may, or may not, be ultimately determined to be "latent factors".

In sum, you have identified a problem, but I don't think you have presented us with a solution.

In sum, it is better to respect the AVAILABLE model test EVIDENCE than to pretend disregarding such evidence constitutes a solution! C&L had the evidence, and many other authors have the evidence – it is time to respect the available evidence.

Reviewer: 2
Comments to the Author

Essentially, all models are wrong, but some are useful (Box & Draper, 1987, p. 424).

Disregarding EVIDENCE that a configural model is problematic is scientific blasphemy. Evidence of model misspecification demands diagnostic investigation and discussion, not disregard. If we have evidence a model is problematic, we have evidence we can pursue. If we do not have evidence a model is problematic, it would take a god to assuredly assert that model's ultimate merit.

Now, onto a review of ORM Ms. No. 15-0075 "Improving Measurement Invariance Assessments: Correcting Entrenched Deficiencies in Testing." As a preamble, I must say that I was reasonably insulted being asked to review a paper that was submitted in such egregious violation of formatting requirements for this journal (APA Version 6). I don't know whether this reflects the authors' ignorance or arrogance, but I hope it is the former as that is ameliorable. Having said that, understand that my remaining comments are independent of this observation.

I know that the word "or" obscures the possibility of options three, four and five. I hope that you as reader read beyond the lines of purveyed options.

The authors address an inconsistency in model selection strategy in practice whereby the goodness-of-fit of the measurement model is evaluated against some model goodness-of-fit index (GFI) such as the Tucker-Lewis index, SRMSE, RMSEA, etc. (and ignoring the fit of the measurement as assessed by the $\chi 2$ statistic), and then proceeding to conduct more specific $\Delta\chi 2$ tests to compare alternative models for the purpose of model selection. These shifting standards are due criticism, but no defensible solutions are offered in this paper. More specific comments are as follows:

Yes there is inconsistency in disregarding $\chi 2$ while attending to differences in $\chi 2$. Unfortunately, disregard of evidence of configural model problems is devastating, and not a mere inconsistency. The "defensible solution" is to pay attention to EVIDENCE of configural model ill fit.

1. It seems that your argument rests on the premise that rather than assessing model GFI that we should instead be "requiring consistency with the world's causal structure" (p. 4). I'm not sure what the "world's causal structure" really is, but I'm presuming that you mean something akin to the True Model. If so, then this is a laudable goal to which we should aspire if we only had access to the True Model.

We can aspire to attain a true model we do not currently possess – such is the nature of scientific research.

Since I think that no structural equation modelers are gods, I also think that none have the requisite god's eye view on things to effect this enterprise effectively.

It is nice to see that the reviewer does not grant Box & Draper sufficiently god-like status to warrant their claim that "all models are wrong" – even in the absence of evidence regarding all models. Scientists are not gods – they are people who respect evidence, and strive for consistency with the evidence. Our assessments of measurement invariance will be more "effective" if we attend to the test evidence than if we do not attend to the available test evidence!

So apparently as a surrogate, you suggest a requirement that configural models' fit approximate that which would be expected on the basis of sampling error alone (i.e., a statistically non-significant $\chi 2$ statistic).

Yes EVIDENCE begins where sampling variability ends.

But as you surely know, we've been wrestling with the dilemma that when sample sizes become defensibly large to consider (N-1)*FML as asymptotically distributed as $\chi2$, then virtually all models are rejected statistically.

There exist structural equation models having no evidence against them. This reviewer seems inclined to disregard EVIDENCE of problems rather than struggle to resolve the problems by appropriately altering their model and corresponding theory. There is NO dilemma in requiring that researchers attend to EVIDENCE their model is problematic.

Of course, this dilemma is what is responsible for the proliferation of the great variety of GFIs we now see in the literature, many of whom are reasonably sample size-independent. So my first main point is that you're no less caught up in this dilemma that any of the rest of us.

False. Using $\chi2$ to differentiate between whether or not the data is beyond what might be reasonably attributable to chance sampling variations is NOT subject to the same dilemma as the GFIs because the GFIs do not address what constitutes EVIDENCE as opposed to random variation.

2. One suggestion you have (p. 5) is to use modification indices to diagnose model misspecifications. The logic of this approach goes back to the mid-1980s (Saris, Satorra, & Sörbom, 1987) and is intuitively appealing until one realizes that (a) it doesn't work (MacCallum, 1986),

Modification indices often do not work but they are not guaranteed to not-work. My sentence that mentions modification indices reports two features that hamper the modification indices.

(b) as you point out, it can capitalize on chance,

And I continued by saying the modification indices are also unable to address the "more difficult" task of correcting a wrong number of latents. The most devastating problem with the modification indices is that they capitalize on NON-chance. A discussion of the multiple weaknesses of modification indices is tangential to the focus of this article.

and (c) it suffers from the same dilemma as use of the $\chi2$ statistic for model selection (MIi = (N-1)*FD, where FD is the first derivative of each fixed parameter with respect to FML).

I did not propose $\chi2$ for model selection. I proposed $\chi2$ to determine whether or not the data provides evidence speaking against the specified configural model.

I will say that Saris, Satorra and van der Veld (2009) have proposed a related approach that has shown some promise, but its efficacy has not really been thoroughly evaluated yet. (See Meade, Johnson & Braddy [2008] for an alternative model selction strategy).

I inserted comments pertinent to Meade, Johnson and Braddy, 2008.

3. Then you get into what I suppose is your paradigm for detecting model misspecifications by introducing exogenous variables (sex and age) that are allowed to have direct effects on latent variables' (LVs') indicators as well as indirect effects through the LVs themselves. Your rationale seems to be that if there are significant associations between these exogenous variables and the manifest indicators' uniquenesses then there is specification error because the designated LVs do not fully explain the covariances among their respective indicators. I have several observations here:

The model requires, and not merely allows, the latent variables to appropriately distribute the Sex and Age (exogenous latent variable) effects to the indicator variables. If the C&L work-latent specification is the proper causal specification, the model-required distribution would be consistent with the data. The reviewer almost got the rationale correct, but should have referred to "residual" associations, not just "associations".

a. I fail to see how what type of genitals one has or even what gender one identifies with and the length of time one has been on the planet can serve as a direct or indirect cause to responses on the survey items you describe.

I was inclined to chide the reviewer's failure "to see" sexism and ageism in the work place, but I will instead note that if there were no such causal connections, Sex and Age should end up uncorrelated with the indicators because the model should fit with zero effects connecting Sex and Age to C&L's latents. The data report otherwise, even if this reviewer does not see why.

Even the examples you provide give explanations that invoke more psychological LVs that may be correlated with sex and age but for which of these surrogates are sex and age appropriate indicators?

Yes there may be more latent variables – more than C&L postulated as CAUSES of the modeled indicators – and these additional latents confront C&L's configural model's specification, and question their assessment of measurement invariance.

b. Parenthetically, this procedure resembles what has been referred to as a "disturbance term regression test" of overidentifying restrictions (James, Mulaik, & Brett, 1984; Lance, 1986).

Yes there is a resemblance. And yes the TEST result indicates the C&L model is causally misspecified**.**

c. There should be no expectation that a single LV should explain all of the systematic variance in its indicators.

This is a philosophically contentious issue, but fortunately one that need NOT be addressed in the context of the Sex and Age variables speaking against C&L's model because the ill fit introduced via these additional variables are not systematic variance in a specific latent variable's indicators. The new evidence of ill fit originates in lack of appropriate coordinate between some latent's indicators and the indictors of OTHER variables (the Sex and Age latents).

A basic assumption of the common factor model is that u = s + e, or the unique factor (u) is comprised of a (systematic) specific component (s) and nonsystematic measurement error (e). I should think that it would come of little surprise that a molar indicator such as sex or age might have some relation with the specific components of LVs' indicators and so I just don't see the logical defensibility of your procedure as an approach for the detection of model misspecifications.

The causal foundation of the reviewer's "some relation" is the issue. C&L's model, and other invariance testing models, do not deserve to disregard evidence of problems merely because the authors are unsurprised by evidence that MAY or MAY NOT, originate in the kinds of problems those authors imagine. If Sex and/or Age function through "s" rather than C&L's work latents, that demonstrates a logical deficiency in C&L's claims about the latent causes of the work indicators, and hence questions their measurement assessments.

And, as I said in point 1 above, this approach is stuck in the same boat as every other sample size-dependent approach to model selection.

Yes the reviewer said this above, and I responded to this above. But the issue is NOT model selection. The issue is whether or not there is EVIDENCE speaking against the configural model that grounds or initiates invariance testing, and whether researchers must respond to evidence of problems in the configural model's specification. Some people will drown, along with their sinking measurement invariance assessments, because they will not get into an evidence-respecting boat.

4. You reiterate in your Discussion section that "Faithful modeling of the worldly causal structures is required to attain adequate and invariance measurements" (I assume this means something like "We have to be fitting the True Model before we can calibrate our measurements.') and "Notice the problematic focus on "fit" rather

than whether or not there is evidence the model is improperly structured" (How can we know whether the model is improperly structured other than by assessing model fit?).

You can do better by moving from "assessing model fit" to respecting and attending to TEST evidence of model problems. Diagnosing what is problematic about a test-failing model is very different than pointing to fit-indices (which disregard some detectable ill fit) as a way to obscure/hide the evidence that the model requires improvement.

Minor, but annoying things:
5. The authors' use of quotation marks is bewildering.

Agreed, and several were corrected.

6. "Latent" is an adjective, not a noun.

Latents and indicators are variables.

7. p.3, l. 1: "error variance" is imprecise. I think you mean "uniquenesses" here.

"measurement error variance" is more SEM-precise than uniquenesses.

8. p. 5, l. 12 from bottom: "p = 0000." Nothing occurs with zero probability.

I did not change/alter the program-reported output to correct the reviewer's misreading of zero to four-decimals as being identical to zero.

9. p. 6: specification of an all-Y model is mathematically identical to a mixed X and Y model so results should be identical.

The parallel estimates are identical (as reported) but there are additional "results" in the form of additional modification indices, EPC's, etc.

References
Box, G. E. P., & Draper, N. R., (1987), Empirical Model Building and Response Surfaces, New York, Wiley.
James, L. R., Mulaik, S. a., & Brett, J. M. (1982). Causal analysis: Models, assumptions and data. Beverly Hills, CA: Sage.
Lance, C. E. (1986). Disturbance term regression test proce¬dures for recursive and nonrecursive models: Solutions from intercorrelation matrices. Multivariate Behavioral Research, 21, 429 439.
MacCallum, R. C. (1986). Specification searches in covariance structure modeling. Psychological Bulletin, 100, 107-120.
Meade, A. W., Johnson, E. C., & Braddy, P. W. (2008). Power and sensitivity of alternative fit indices in tests of measurement invariance. Journal of Applied Psychology, 93, 568-592.

***I included new comments regarding this article*.**

Saris, W. E., Satorra, A., & Sörbom, D. (1987). The detection and correction of specification errors in structural equation models. In C. C. Clogg (Eds.), Sociological methodology 1987 (pp. 105–129). San Francisco: Jossey-Bass.
Saris, W. E., Satorra, A., & van der Veld, W. M. (2009). Testing structural equation models or detection of misspecifications? Structural Equation Modeling.16, 561-582

Reviewer: 3

Comments to the Author
I have completed my review of ms. 15-0075 titled “Improving Measuremetn-Invariance Assessments: Correcting Entrenched Deficiencies in Testing.” I would like to thank the author(s) for sharing their work. I appreciate the opportunity to serve as a reviewer. I firmly believe the topic of this paper would be of substantial interest to readers of the journal and I certainly agree with the idea that we should always be seeking to improve our methodological practices. That said, however, I do have some concerns with the current paper that I believe may limit the degree to which it can make a meaningful contribution to the organizational science literature.

1. One of my biggest concerns with the current paper is a disagreement with the fundamental premise regarding expectations of what can be taken as evidence of model fit.

Yes, I attend to evidence pointing toward model misspecification, while the reviewer’s philosophy is to be less attentive to the evidence.

I was intrigued with the notion that one might conduct invariance testing using a fuller structural model, but the underlying logic for doing so requires significantly greater development

The greater development is available in the wider (non-factor model) structural equation modeling literature (e.g. Hayduk, 1996).

and I fear might move the field backwards in at least one respect.

… in one as yet unspecified respect. An unspecified concern requires no response.

2. I found the language used in the paper to be unnecessarily combative and condescending in terms of previous work in this area.

I slightly reduced the pointedness of some comments, but I hope some combativeness still shows through. I expect readers considering these Organizational Research Methods reviews and my comments will sense some fundamental philosophical points worth contending.

The tone of this paper suggests that there is only a single way to conduct and interpret model fit (i.e., chi-square) in tests of measurement invariance and SEM more generally.

That is close to “the tone” and intent – but notice the reviewer’s mistake of referring to “model fit” rather than to the properness of the configural model’s causal specification. There are many ways to address fit, but only one reasonable way to address model properness.

If that is the position, I think more evidence is required to support such statements.

The evidence is available in the wider structural equation modeling literature.

Most importantly, this evidence has to be more than a simple assertion. That is, I think readers will want and need to see concrete references for such statements that speak to the ills of alternative fit measures such as the CFI, TLI, etc.

I do not reference these, or other specific fit indices, and instead I referenced publications displaying the weakness of ALL fit indices.

3. I question the statement on page 4 that, “Model testing asks whether or not the current data provide evidence of inconsistency between the model’s structure and the world’s structure….” I completely disagree.

I urge the reader to consider this reviewer statement very carefully. Does, or does-not adequate measurement REQUIRE consistency with worldly features? I claim measurement is nonsense unless the measurements display some specific coordination with the world.

Such a statement implies that one's data represents the world.

The data comes from the world, and the data stand as the available representations of the world, even if we do not unerringly know the structure of the world that produced the data.

I don't see how such an inference can be supported.

I see a claim to measurement as nonsense (not sensible) unless the world contributes to the "measurements".

For me, this calls into question the entire premise of the paper, as well as the fundamental conceptual foundation upon which all arguments are based.

Clearly there are philosophical differences a play here – but the matter is not playful, it is deadly serious and concerns what constitutes measurement in scientific research. Too bad the reviewer did not articulate any specific "measurement without a corresponding world" philosophy for me to counterbalance.

4. Another rather significant concern I have with the paper is the suggestion that we should be working with single indicator models.

***The article recommends use of the few best indicators. If a researcher can't even get two reasonable indicators, they may have no choice other than to use a single indicator*.**

The reason we prefer multi-item scales is for reasons of reliability and more adequate coverage of the underlying latent space.

Seeking a proper model causal specification is seeking VALIDITY, not mere "reliability". Each factor only has one dimension (no matter how many indicators it has), and the factor does not cover a latent space – it covers a latent line. Using several latents to cover a set of items permits superior "coverage of the underlying latent space". The article recommends using as many latents as are required to appropriately causally cover the latent space. Readers interested in this topic might see Hayduk and Littvay (2012).

To suggest that we should take several steps backward and work with measures that rely on fewer indicators (i.e., 1) strikes me as ultimately counterproductive (point made on p. 7 right before the Summary section).

The point made there (before the Summary section) was that some of even the pairs of C&L indicators displayed inconsistencies that keep those indictors from displaying adequate measurement, let alone invariant measurement. If a researcher has two or more ADEQUATE indicators, use them. If there is available EVIDENCE that even a pair of indicators are problematic, respect the evidence. The current article reports evidence that some pairs of C&L indictors do not work well together, despite C&L's claims of measurement invariance. It would constitute a step forward if this reviewer acknowledged and responded to the evidence that some pairs of indicators do not function as if they were responding to a single-common underlying latent cause. If this reviewer wishes to challenge the use of the few best indicators, she/he should provide a response to Hayduk and Littvay (2012).

5. In the first paragraph, I think more support is needed for some assertions. In particular, on what basis would it be the case that factor analysis downplays model testing? Is this a reference to EFA exclusively or is CFA also swept into this. If CFA is included, then I think this statement is simply untrue. There is a fairly rich history of testing alternative measurement models. If only EFA, I still believe more should be said given that EFA is often characterized by testing various models for interpretability.

That paragraph refers explicitly to weak factor analytic "model testing", not "interpretability". I believe the reviewer's statement about CFA is "simply untrue". Notice, for example, that C&L tested the difference between their models (one interpretation of the reviewers "testing alternative measurement models") even though C&L did NOT do an adequate job of testing their models. Vandenberg and Lance report regarding measurement invariance that "although rarely tested, these assumptions are routinely

and straightforwardly testable as extension to the basic CFA framework" (2000:6). They clearly report that while CFA makes things TESTABLE, the testing is "rarely" done.

6. Related to the point above, the latter part of the second paragraph asserts that measurement invariance (MI) is not characterized by model testing. This seems inconsistent with provided references (e.g., Vandenberg & Lance). I think this makes the message more ambiguous.

According to Vandenberg and Lance: "measurement invariance is rarely tested in organizational research" (2000:4), and "although rarely tested, these assumptions are routinely and straightforwardly testable as extensions to the basic CFA framework" (2000:6). Similar statements appear on their pages 2000:9,11. Their Table 1 provides multiple references speaking to assessing configural invariance, but the word testing does not even appear in this column, and the related text does not clearly require model testing rather than model-difference-testing. Furthermore, notice the definite switch in Vandenberg and Lance from the hundreds of times testing is mentioned in the early parts of their article (where it is clear that their statements become unconvincing if the model is the wrong structural model) to the use of "model fit" (page 43) – where concern for a structurally-wrong but close-fitting model is NOT addressed. Vandenberg and Lance did acknowledge that even back in 2000 "the general issue of fit ... is … in a state of evolution" (42-43), and in reference to their suggested standards, that "no researcher should adopt these standards without first examining the current literature" (2000:44). Vandenberg and Lace seemed unaware of the Hayduk and Glaser (2000) discussion of structural equation model testing. And subsequently, in 2007 Personality and Individual Differences (42(5)) a target article (by Paul Barrett) which was followed by multiple commentaries, questioned the use of fit indices, as opposed to model testing. The testing arguments were further clarified in Hayduk (2014b). The paragraph the reviewer referred to does not cite the Vandenberg and Lance article, but does cite Hayduk and Glaser (2000) and Hayduk (2014a and b). I added an additional reference from the 2007 special issue of Personality and Individual Differences.

7. I know that most readers of a paper like this will already be relatively well-versed in MI, but I do think the description provided on page 2 could be even more explicit. Further, the ideas presented regarding how MI has traditionally been viewed/conducted could benefit from much greater detail regarding techniques and findings.

Rather than extending the discussion, I inserted a reference to where more detailed discussion is already available – namely to Vandenberg and Lance, 2000.

8. Given the underlying intensity of the present argument regarding the 'ideal' approach to conducting MI (and SEM more generally), I was surprised that there wasn't more said about using ordinal (Likert) level data. Are there no concerns that using such data may lead to inappropriate inferences?

Addressing the issue of ordinality would distract from the main focus of the article. If the reviewer, or anyone, wishes to claim C&L's model failures are likely due to the ordinality of the data, they can check this for themselves because the data are publicly available.

9. Somewhat related to the previous point, I don't understand why different analytic choices were made if the desire was to directly compare results with those reported by Chueng & Lau. I can appreciate that there may have been differences to sample size, but why not keep analyses as comparable as possible?

I did keep the analyses as comparable as possible – without muddying the discussion by introducing distracting bootstrapping procedures.

10. The author(s) need to make a much more compelling case that the practice of MI (at least those that follow logic such as that proposed by Vandenberg & Lance) has led of erroneous MI reports.

The article provides a compelling case that C&L's discussion of measurement invariance was erroneous. Some evidence from Vandenberg and Lance is now also provided.

I know this is a direct statement made in the paper and that there are several places that make similar assertions, but there should be more evidence than a mere assertion. I don't believe sufficient evidence has been provided to fundamentally question these practices.

Yes the relevant statements were repeatedly made. The relevant model test results are also presented – but it seems that: highly significant evidence of model-data inconsistency; extension to additional variables that also display highly significant evidence of inconsistency between the data and C&L's model; and highly significant failure of the model using even the very best of C&L's indicators; does not constitute "sufficient evidence" for this reviewer.

11. I found the Abstract to be rather lengthy and not particularly smooth or efficient. I think simplicity would be preferable.

I have revised the abstract slightly – and hope the listing aids simplicity.

===

(***The submitted manuscript.***)

Improving Measurement-Invariance Assessments: Correcting Entrenched Deficiencies in Testing

Abstract

Factor analysis historically focused on measurement while path analysis employed observed variables as though they were error-free. When factor- and path-analysis merged as structural equation modeling, factor analytic notions predominated the measurement discussions – including assessments of measurement invariance across groups. The factor analytic tradition fostered disregard of model testing and consequently entrenched this deficiency in invariance assessments. Applying contemporary model testing requirements to the so-called "configural model" in invariance assessments will improve future assessments but a substantial backlog of deficient assessments will remain to be overcome. This article summarizes the issues, demonstrates the problem using a recent example, and illustrates an enhanced assessment strategy. Employing the few methodologically and theoretically best indicators, rather than multiple indicators, of latent variables will increase the likelihood of achieving properly causally specified structural equation models.

Keywords

Invariance, Factor analysis, Testing, Close fit, Structural equation model

Background

Structural equation models meld a measurement "model" composed of the causal connections between latent and observed variables, to a latent-level "model" composed of causal connections between the latent-level variables. The measurement and latent model-components tended to be viewed as distinct because the measurement model-segment historically developed from the factor analytic tradition (Thurstone, 1947; Harman, 1967; Lawley & Maxwell, 1971) while the latent level model-segment followed the path analytic tradition (Wright, 1921, 1934; Blalock, 1964; Duncan, 1975; Heise, 1975). These model-segments can be appropriately and relatively easily statistically combined, but the factor tradition of downplaying and evading model testing conflicted with the path analytic tradition of attentive model testing

Structural equation model measurement specifications deserve the same careful scrutiny as latent level interconnections because the latent level cannot function appropriately without proper measurements, and measurement is not assured unless the supposedly-measured latents function appropriately (Hayduk & Glaser, 2000; Hayduk, 2014a,b). The question of measurement invariance arises when researchers consider whether an indicator item measures the same thing in different contexts – whether different countries, different religions, or with different languages. Unfortunately, measurement invariance assessments remained a bastion of resistance to model testing, which taints the resultant assessments. This article explicates the concern for configural model testing, and illustrates the resultant problems by reconsidering and extending some recent advice regarding measurement invariance assessments.

Measurement invariance assessments frequently begin with a factor-structured model that is progressively constrained by adding between-group equality constraints on the "loadings", measurement error variances, and measurement intercepts. Changes in "loadings" (the causal actions leading from the latents to indictors) are usually granted priority because differences in loadings directly signal differences between the observed indicators and the underlying latents that are supposedly measured. If an indicator is more strongly responsive to a latent in one group than another, the prima facie concern is that this signals a change in the causal source of the indicator – something different may be being measured in the two groups, and hence the measurement may be varying. Parallel comments apply to measurement error variances, intercepts, and other model coefficients. Consequently, testing the tenability of between-group

equality constraints on loadings, and other model coefficients, underpins assessments of measurement consistency or invariance.

Investigating between-group constraints is reasonable as long as historical factor-analytic inattention to model testing has not undermined the very foundation of this approach. A factor-structured model, called the configural model, often constitutes the initial model in the invariance testing process. The configural model typically results from prior exploratory factor analyses which place each indicator under a specific factor. The clustering of indicators under latent factors is conveyed by requiring zero "cross loadings" between latents and the indicators of "other" factors, and zero measurement error covariances (corresponding to the presumption of "statistically independent" errors) within each group. The basic configuration that grants the name "configural model" is the identical clustering of indicators under latent factors in the different groups. The configural model has no between-group constraints on the estimated coefficients, so the loadings, error variances, and other estimates may differ between the groups, though the placement of the loadings retains the same configuration in both groups.

The current issue is whether or not this configural model must be carefully tested. The perspective taken here is that the configural model must be carefully tested – which conflicts with the entrenched practice of treating significant inconsistency between the data and configural model as "acceptable" or "tolerable" at the initial stage of invariance assessments (e.g. Byrne, Shavelson & Muthen, 1989; Cheung & Rensvold, 1999; Vandenberg & Lance, 2000; Cheung & Lau, 2012). Defending measurements as invariant on the basis of consistency between groups is nonsense if the model's structure is inconsistent with the world's causal structure. If the configural model's structure does not correspond to the world's causal structure, asking about invariance between the groups is asking whether the groups agree in their ***mis***representation of the connections between the indicators and the underlying latents! Measurement invariance makes sense not merely because the groups agree with one another but because there is between-group agreement *as well as consistency between the model and the measured world's causal structure*.

Data and Procedures

To illustrate both the problem and a helpful alternative, we consider the example discussed by Cheung and Lau (2012) which used the Work Orientations data for residents of the United States and Great Britain published in 1989 by the International Social Survey Program (ISSP, 1989) which is publically available. SPSS 22 (IBM, 2013) was used for calculation of the basic indicator statistics, and maximum likelihood estimates from covariance input were obtained via LISREL 9.1 (Joreskog & Sorbom, 2013). The survey questions providing the indicators, and their means and standard deviations are provided in Table 1.

Analysis and Context

The configural model initiating the testing sequence investigated by Cheung and Lau (2012) (hereafter C&L) is a factor model having four indicators of each of three latent variables: quality of *Job Context* (η_1), quality of *Job Content* (η_2), and quality of *Work Environment* (η_3). This

model (for one group) is depicted in Figure 1. The configural model is the Figure 1 model estimated simultaneously but separately for Great Britain (GB) and the United States (USA), with no constraints between the groups, so all the "loadings" and other model coefficients receive a unique estimate in each group. For clarity we employed a 1.0 loading for the first indicator in each indicator set to scale the corresponding latent variable. C&L focused on bootstrap procedures incorporating less-common scaling options, but these features of their analyses are tangential to our concern – which is the testing of the basic or initial configural factor model.

C&L say: "As good model fit is a prerequisite for meaningful interpretation of BC bootstrap confidence intervals, it is necessary to ensure that the configural invariance model shows adequate model fit" (Cheung & Lau, 2012:172-173). C&L's concern for the adequacy of the configural model is laudable, but this statement nonetheless remains problematic. The C&L reference to bias-corrected bootstrapping is moot and subordinate to our concern. The problem centers on "adequate model fit". It is important to differentiate between model "fit" and "model properness" because seriously causally misspecified/wrong factor models can fit. Hayduk (2014a), for example, illustrates that it is sometimes possible for a one factor model to *perfectly fit* data generated by three real-world latent factors. It would be nonsense to claim adequate or invariant measurement of "one" factor if the real world contains three factors, not one! Given that causally wrong factor models can provide perfect-fit, it should be obvious that more-wildly misspecified models can produce near-fit, or close-fit. The ability of causally incorrect models to nearly-fit makes it unreasonable to employ closeness-of-fit as a foundation for assessment of measurement, and measurement invariance. The appropriate measurement concern is not some indexed amount or closeness of "fit", but whether or not the data are consistent with the specified number of underlying latents and their modeled connections to the indicators (Borsboom, Mellenberg & vanHeerden, 2004).

Model testing asks whether or not the current data provide evidence of inconsistency between the model's structure and the world's structure; fully recognizing that evidence of causal inconsistency disrupts the very basis of measurement. Factor analysis historically focused on close fit, and close fit indices, with intentional disregard for testing the consistency between the model and the available data. C&L continued this effete factor tradition when they required that the "configural invariance model shows acceptable model ***fit***" (2012:173, emphasis added) rather than requiring consistency with the world's causal structure. The switch away from model-properness to model-fit is fundamentally problematic because it pretends measurement could be called adequate and invariant even if the available evidence reports the configural model's causal structure is inconsistent with the world's structure.

Unfortunately, C&L really meant, trusted, and depended-on, *fit* as opposed to respecting evidence of world-model causal consistency or inconsistency. C&L report that their configural model provides χ^2 = 399.6 with 102 degrees of freedom – namely with 51 degrees of freedom in each group. C&L did not report the corresponding probability, though anyone knowing that a χ^2 having many degrees of freedom is nearly normally distributed with mean equal to the degrees of freedom and variance twice the degrees of freedom (so a standard deviation is $\sqrt{2df}$) should not need a χ^2 calculator to determine C&L's configural model's χ^2 is about 20 standard deviations from the mean, and hence has a $p < 0.000001$.

That is convincing evidence of inconsistency between the data and C&L's configural model. This probability informs us that there is essentially no chance that random sampling variations could account for the difference between the available data and the C&L configural model, even after the configural model is supplemented with optimal loading and measurement error variance estimates in each group. To say this another way – there is essentially no chance that the observed data could have arisen via random sampling if the worldly causal forces were structured as in the configural model. And yet another way – something about the configural model's specification must be changed in order to match or correspond to the world's causal structure.

The model might be wrong for claiming three latents exist. If there were four, or more, latents in each country, it would be nonsense to claim the indicators adequately measured three latents! It would be similar nonsense to claim adequate *invariant* measurement, if the available data are inconsistent with three modeled latent factors after constraining the loadings or other estimates to be equal between the groups. In fact, very one of the 24 models with between-group constraints reported by C&L in their Table 6 is highly significantly inconsistent with the ISSP data (Cheung and Lau, 2012:181).

It is strange, but consistent with factor analytic history, that C&L attend to significant increases in χ^2 upon insertion of between-group constraints (where about half the "significant" χ^2 changes are on the order of 10 to 20) and yet they disregard the comparatively huge 399.6 χ^2 for their base configural model. They attend to small χ^2 changes when freely estimated coefficients are constrained to equality between the groups but fail to attend to the huge χ^2 change that accompanied constraining the zero cross-loadings and other features of the configural model to "equality between the groups". They attend to constraints that result in estimates but disregard the stronger constraints that provide specific coefficient values.

One option for investigating what might be wrong with C&L's configural model would be to check the modification indices for cross-loadings and error covariances in the configural factor model, but this approach is hampered by capitalization on chance, and it disregards the more difficult possibility that the configural model may contain the wrong number of latents. We chose the alternative approach of adding new evidence to the discussion by including some possible causes of the three latents C&L postulated. Given that men and women, and the young and old, likely have different work experiences and concerns, Sex and Age likely impact at least some of the work-focused indicators. If the C&L latents are the appropriate causal foundations of the indicators, Sex and Age should only impact the indicators indirectly through those three work latents. The covariances between Sex, Age and the ISSP work indicators constitute new evidence regarding the C&L postulated configural causal structure. If effects leading from Sex and Age to the three modeled work "factors" (now more accurately called work "latents") are unable to account for the covariances between Sex, Age and the specific work indicators, that constitutes evidence that the postulated work latent factors are not the appropriate causal foundations of the indicators. This is an instance of single-indicated latents (Sex and Age) being used to more fully assess multiple-indicated latents/factors, namely C&L's work latent factors (Hayduk, 1996; Hayduk & Littvay, 2012).

We began by attempting to replicate the failure of the C&L configural model with the ISSP data. The resultant χ^2 = 448.4 with 102 degrees of freedom and $p = .0000$, and was similar though not identical to C&L's χ^2. The difference in χ^2 values may be partially due to our analysis having three more respondents in each country than was reported by C&L. C&L report 645 and 820 cases, while we obtained 648 and 823 cases for Great Britain and the USA respectively. (Cheung & Rensvold, 1999 also report 823 USA cases in the ISSP data, so C&L's slightly reduced *N*s seem inexplicable.) The difference in χ^2 values may also be partially due to our using maximum likelihood in LISREL rather than C&L's bootstrapping in Mplus. Whatever the reason for the difference in χ^2 values, both agree that the C&L configural model is definitely inconsistent with the ISSP data. Furthermore, the problems with the configural model's specification seem spread throughout the model rather than being "localized" – as evidenced by 9 and 11 (of the 24 possible) cross-loadings in the GB and US models having modification indices exceeding 4.0 (respectively).

Sex and Age were then added to the model as correlated single-indicated exogenous latent variables (each with 2% measurement error variance) that were permitted to influence all three work latents (as in Figure 2, for one group/country). No constraints were entered between the countries, so each country received its own estimates for both the new and original model coefficients. This specification ensures the work-measurement section of the model closely parallels the C&L configural model, though the new model is a full structural equation model, and not just a factor-structured model.

It was unsurprising that the Sex and Age supplemented model fails (χ^2 = 680.2 with 138 degrees of freedom and $p = .0000$) because this model's structure cannot rectify the ill fit previously observed among the work indicators (where χ^2 was 448.4), because there are no direct connections between the new variables and the work indicators. (The C&L three work latents' loadings, variances and covariances continue to constitute the only explanation for relationships among the work indicators.) Nonetheless, the substantial increase in χ^2 constitutes important news because this increase originates primarily in the work indicators' covariances with Sex and Age. The 18 new degrees of freedom in each group come from attempting to explain the 24 new covariances (between Sex, Age, and the 12 work indicators), with the six new effects leading from the Sex and Age latents to the work latent "factors". The substantial jump in χ^2 is a sign that the six latent connections between Sex, Age and the work-latents are insufficient to account for the 24 covariances between Sex, Age and the work indicators. The standardized covariance residuals for 10 and 13 of these 24 covariances exceed 2.0 in the GB and US groups respectively, and hence would be significant if tested individually. If the configural model's work latents had constituted the correct causal foundations of the work indicators, those three latents would have been able to appropriately distribute the causal impacts of Sex and Age to the work indicators. The multiple inconsistencies between the new covariances and the covariances required to conform to C&L's configural work-latent work-indicator specification, clearly report that the work latents in the C&L configural model are inconsistent with the new evidence provided by the covariances between Sex, Age, and the work indicators.

Another perspective on this is obtained by specifying Sex and Age in an all-η model (so the Sex latent and Age latent become η_4 and η_5). This produces the same estimates and ill fit of the work-indicators' covariances with Sex and Age as reported for the Figure 2 model above, and in

addition provides modification indices corresponding to potential effects leading directly from Sex and Age to each specific work indicator. Of the 24 potential "loadings" leading from the Sex and Age latents to the work indicators, 10 and 13 of the modification indices exceed 4.0 for GB and the US respectively. (As in the basic C&L configural model, with no Sex or Age, many of the cross-loadings from the work-latents to the work-indicators continued to have modification indices exceeding 4.0.)

Each real direct Sex or Age effect to a specific work indicator challenges C&L's presumption that three work latent-factors constitute the causal foundations of the work indicators. Each direct effect from Sex or Age to a work indicator reports that the C&L basic configural model is wrong because such effects are inconsistent with the configural model's causal structure. Such effects would make Sex and Age additional latent common-causes of the work indicators – via the direct effects and indirect effects of Sex and Age functioning through the work latents. Thus both the covariance inconsistencies, and the modification indices connecting Sex and Age to the work indicators, report that the work latent "factors" in the C&L configural model are problematic, and that more, and/or different, latents are involved in producing the work-indicators.

So both the previously available evidence from within the set of work indicators themselves (as reported by C&L), and the new evidence from attempting to connect the work indicators to Sex and Age as exogenous causes, speak against the causal structuring of the work-latents in C&L's configural model. But there remains the possibility that only the work indicators surviving C&L's additional invariance investigations might fare better than the full set of indicators. C&L's subsequent investigation of invariant intercepts might have coincidentally weeded out some covariance-problematic indicators, leaving only indicators appropriately modeled by the C&L work latents.

This was investigated by setting up a model similar to the Figure 2 model, but employing only the work indicators C&L report as additionally displaying intercept, or scalar, invariance. C&L report the relevant indicators: for Work Content as y_5, y_6, and y_8; for Work Environment as y_9, y_{10}, and y_{12}; but left Work Context to be indicated either by the pair y_1 y_4 or the pair y_2 y_3 "based on theoretical interpretation and the research question" (Cheung & Lau, 2012:178). Using the y_2 y_3 pair for Work Context; y_5, y_6, y_8 for Work Content; and y_9 y_{10} y_{12} for Work Environment, along with Sex and Age, results in a model that continues to be severely inconsistent with the covariance data (χ^2 = 287.0 with 54 degrees of freedom and $p = .0000$) and displays the same general pattern of inconsistencies reported above for the full set of work indicators. Using the y_1 y_4 pair produces similar results but with additional evidence that the y_1 y_4 indicators in the GB group are too inconsistent with one another to support any reasonable covariances among the work latents. Collectively, these observations convincingly report that appealing to intercept-consistency cannot dispel or overcome the covariance-inconsistencies introduced by overlooked configural model misspecification.

We estimated one additional model employing Sex, Age, and two indicators of each work latent: y_2 and y_3 for Work Content, y_5 and y_6 for Work Context, and y_9 and y_{10} for Work Environment. This model also fails to match the covariance data (χ^2 = 132.9 with 12 degrees of freedom and $p = .0000$) and displays the same scattered pattern of residual ill covariance fit, and numerous substantial modification indices connecting Sex, Age, as well as the work latents (via

cross-loadings) to the work indicators. This instructs us that even pairs of indicators can detect problematic configural models, and implicitly instructs us that appropriate causal model specifications may require some single-indicated latents. The diagnostics in this paired-indicators model became much more focused and specific than the diagnostics for the models having more indicators, and hence paired indicators may helpfully accentuate specific theoretical or methodological issues, concerns, and options. This suggests it may be useful to begin invariance testing with the few best indicators, rather than beginning with “multiple” indicators.

Summary and Discussion

The preceding results prompt several observations. First, if a researcher intends to investigate the invariance of measurements between groups, the basic model structure – the configural model initiating the invariance assessment – must be consistent with both groups’ data. Evidence of inconsistency between the configural model and either group’s data may be signaling that the model contains incorrect latents. Incorrect latents render all measurements, including invariance of measurements, dubious because measurement is meaningless if a modeled latent has no corresponding worldly feature (Borsboom, Mellenberg, & vanHeerden, 2004).

Second, the configural model initiating invariance testing need not be factor structured. It is reasonable to start with a full structural equation model that includes exogenous variables like Sex and Age. Indeed, it seems preferable to begin with a configural model whose latent structure is consistent with the researcher’s theory, their methodological understanding, and the data. Measurement and measurement-invariance assessments should be integrated with latent level structural understandings. Latents are known through their indicators – the basic factor claim – but they are not *only*-known through their indicators. Latents are also known through the latent-level causal structures in which they participate – like Sex and Age structures. A substantial but avoidable factor-bias, and corresponding latent-theory weakness, underlies routinely initiating measurement invariance testing with a factor-structured configural model.

Third, it seems self-destructive to begin invariance testing with multiple indicators, whenever it is likely to be difficult to obtain even a few reasonably-functioning indicators. Recall the failure of the configural model having just two indictors per work-latent, and that the diagnostics became noticeably clearer in the context of indicator pairs.

Fourth, researchers are urged to think causally about *all* the variables in their model. The covariances within each set of indicators, and between diverse sets of indicators, result from productive, impactful, consequential, effects in the real world. Faithful modeling of the worldly causal structures is required to attain adequate and invariant measurements. If the model’s structure is inconsistent with the indicators’ worldly causal milieu, the very notion that the indicators are measures is rendered dubious. Our models must structurally mirror the worldly causal impacts if they are to testify to the adequacy of measures of worldly features (Borsboom, Mellenberg, & vanHeerden, 2004, 2003; Borsboom & Mellenbergh, 2002).

In the C&L context, clear causal thinking would prompt researchers: to consider whether the ***same*** basic latent variable (Job Content?) *causes* workers to feel both that their job helps other people *and* that their job is interesting; and to consider whether doing hard physical work is

caused by the ***same*** thing that causes unhealthy work conditions, like exposure to diseases or dangerous chemicals (Work Environment?); and whether a job being "secure" (like a government job?) is produced by the same underlying *cause* that makes jobs have flexible working hours (Job Context?). In the context of invariance assessments, the causal considerations should include the possibility of context-dependent causal impacts. For example, the above models represented Sex as having effects on the work latent variables, whereas proper measurement might require modeling statistical interactions because the relevant causal effects may differ between the sexes. For example, physically demanding work and unhealthy work conditions might be embedded in different causal networks for males than females due to differences between typical male and female work environments. Our focus on statistical matters means that we, like C&L, are limited in the depth to which we can investigate the work indicators' methodology and causal embeddedness. But the important point remains that proper causal specification, however complex, is a mandatory foundational requirement for measurement invariance assessments. Understanding a latent factor as "something common to the items" is likely to be too causally imprecise to support a meticulously-causal configural model.

Cheung and Lau (2012) are not alone in disrespecting evidence of misspecification of the configural model initiating measurement invariance assessments. Indeed, there is a long and inglorious history of disrespect of configural model testing among even the oft-cited foundational papers. Byrne, Shavelson and Muthen, for example, say that "A nonsignifiant χ^2 (or a reasonable fit as indicated by some alternate index) is justification that the baseline models fit the observed data" (1989:457). Notice the problematic focus on "fit" rather than whether or not there is evidence the model is improperly causally structured. Byrne, Shavelson and Muthen subsequently report factor models whose χ^2 values are more than 14 and 41 standard deviations from the mean, and hence whose p values are < 0.000001, and even after 5 different modification-index prompted changes in each group, both groups remain significantly inconsistent with the data – one with so small a p value that it could only be reported as 0 by two different web calculators. The model's inconsistency with the data, even following data-prompted revisions, directly contradicts Byrne, Shavelson and Muthen's claim that their baseline model constitutes a "reasonable representation of the data" (1989: 460).

A decade later, Vandenberg and Lance said: "Overall model fit refers to evaluating the ability of the a priori model to (at least approximately) reproduce the observed covariance matrix" (2000:43), where the laxity of "at least approximately" is obvious, and where the concern is again inappropriately expressed as "fit" rather measurement's requirement of proper model causal specification. And nearly yet another decade later Schmitt and Kuljanin reported that a configural model with $\chi^2 = 1183.86$ and df=174 whose p is reported as < 0.01 but that is also < 0.000001 "was accepted because considerable prior research confirmed the discriminant and convergent validity of these items" (2008:218) – as if clear evidence of problems in the current model's specification could be justifiably disregarded because it would conflict with others' claims! Schmitt and Kuljanin acknowledge that their review of more than 80 recent measurement invariance studies discovered that what authors "accepted as adequate evidence of configural invariance varied considerably across studies" and that what "constituted adequate fit was invariably subjective" (2008:212) – again notice the misguided emphasis on "fit", which easily but inappropriately translates into fit-indices rather than concern for the causal properness of the

model. The widespread fit-propelled disregard of evidence of model causal misspecification has undoubtedly led to more than a few optimistic-yet-erroneous measurement invariance reports.

Conclusion

Ensure that a theory-appropriate and methods-appropriate causal configural model is consistent with your indicators before moving to any other steps in measurement invariance assessment.

Declaration

The author declares no potential conflicts of interest with respect to, and received no financial support for, the research, publication, or authorship of this article. The use of public data obviates the need for ethical review.

References

Blalock, H. M. Jr. (1964). *Causal Inference in Nonexperimental Research.* Chapel Hill: University of North Carolina Press.

Borsboom, D., Mellenbergh, G. J., & vanHeerden, J. (2004). The concept of validity. *Psychological Review, 111*(4), 1061-1071.

Borsboom, D., Mellenbergh, G. J., & vanHeerden, J. (2003). The theoretical status of latent variables. *Psychological Review, 110*(2), 203-219.

Borsboom, D., & Mellenbergh G. J. (2002). True scores, latent variables, and constructs: A comment on Schmidt and Hunter. *Intelligence, 30*, 505-514.

Byrne, B. M., Shavelson, R. J., & Muthen, B. (1989). Testing for the equivalence of factor covariance and mean structures: The issue of partial measurement invariance. Psychological Bulletin, 105(3), 456-466.

Cheung, G. W., & Lau, R. S. (2012). A direct comparison approach for testing measurement invariance. Organizational Research Methods, 15(2), 167-198.

Duncan, O. D. (1975). Introduction to Structural Equation Models. New York: Academic Press.

Harman, H. H. (1967). Modern Factor Analysis, 2nd edition. Chicago: University of Chicago Press.

Hayduk, L. (1996). LISREL Issues, Debates, and Strategies. Baltimore: Johns Hopkins University Press.

Hayduk, L. (2014a). Seeing perfectly fitting factor models that are causally misspecified: Understanding that close-fitting models can be worse. Educational and Psychological Measurement, 74(6), 905-926.

Hayduk, L. (2014b). Shame for disrespecting evidence: The personal consequences of insufficient respect for structural equation model testing. BMC Medical Research Methodology, 14:124, 1-10.

Hayduk, L., & Littvay, L. (2012). Should researchers use single indicators, best indicators, or multiple indicators in structural equation models? BMC Medical Research Methodology, 12:159, 1-17.

ISSP Research Group (1991): International Social Survey Program: Work Orientations I – ISSP 1989. GESIS Data Archive, Cologne. ZA1840 Data file Version 1.0.0, doi:10.4232/1.1840.

Joreskog, K., & Sorbom, D. (2013). LISREL 9.1 March 2013. Skokie, IL, USA: Scientific Software International.

Lawley, D. N., & Maxwell, A. E. (1971). Factor Analysis as a Statistical Method, 2nd edition. London: Butterworth & Co.

Schmitt, N., & Kuljanin, G. (2008). Measuring invariance: Review of practice and implications. Human Resource Management Review, 18, 210-222.

IBM (2013). IBM-SPSS 22. Armonk, NY, USA: International Business Machines Inc.

SEMNET. The structural equation modeling discussion network. http://www.aime.ua.edu/cgi-bin/wa?A0=SEMNET hosted by the Seebeck Computer Center at The University of Alabama.

Thurstone, L. L. (1947). Multiple Factor Analysis. Chicago: University of Chicago Press.

Vandenberg, R. J., & Lance, C. E. (2000). A review and synthesis of the measurement invariance literature: Suggestions, practices, and recommendations for organizational research. Organizational Research Methods, 3(1), 4-70.

Wright, S. (1921). Correlation and causation. Journal of Agricultural Research, 20(7), 557-585.

Wright, S. (1934). The method of path coefficients. Annals of Mathematical Statistics, 5, 161-215.

Table 1: The ISSP Work Indicators

Indicator Wording	Designation here, and in Cheung & Lau	ISSP	Great Britain Mean	Great Britain Std. Deviation	United States Mean	United States Std. Deviation
My job is secure	y_1	V59	2.46	1.073	2.09	.977
My income is high	y_2	V60	3.38	.956	3.22	1.009
My opportunities for advancement are high	y_3	V61	3.29	1.030	3.00	1.115
My job has flexible working hours	y_4	V67	3.16	1.218	2.82	1.192
My job is interesting	y_5	V63	2.11	.852	2.12	.967
I can work independently	y_6	V64	2.08	.838	2.09	.965
In my job I can help other people	y_7	V65	2.28	.951	2.07	.913
{how often}...are you bored at work?	y_8	V71	2.19	.937	2.24	.953
{how often}...do you have to do hard physical work?	y_9	V69	3.46	1.253	3.48	1.186
{how often}...do you work in dangerous conditions?	y_{10}	V72	4.09	1.115	3.97	1.164
{how often}...do you work in unhealthy conditions?	y_{11}	V73	4.06	1.100	4.14	1.042
{how often}...do you work in physically unpleasant conditions?	y_{12}	V74	4.16	1.067	4.10	1.022
Sex	x_1	V85	1.44	.496	1.47	.499
Age	x_2	V86	39.31	11.463	38.62	11.914

ISSP = International Social Survey Program 1989. We refer to Great Britain (rather than C&L's United Kingdom) because that is the designation used in the ISSP. N=648 Great Britain, 823 United States. Items y_1 to y_7 had lead-in: "For each statement about your main job below, please circle one code to show how much you agree or disagree that it applies to your job. 1=Strongly Agree, to 5= Strongly Disagree, 8=Can't Choose." Items y_8 to y_{12} had lead-in: "Now some more questions about your working conditions. Please circle one code for each item below to show

how often it applies to your work. 1=Always, 2=Often, 3=Sometimes, 4=Hardly Ever, 5=Never, 8=Can't Choose". y_8 is reverse coded. Sex: 1 = male, 2 = female. Age: in years. Only those working 10 hours per week or more for pay responded to the above questions. According to Cheung and Lau: $y_1$to y_4 indicate quality of **Job Context**; $y_5$to y_8 indicate quality of **Job Content**; and $y_9$to y_{12} indicate quality of **Work Environment**.

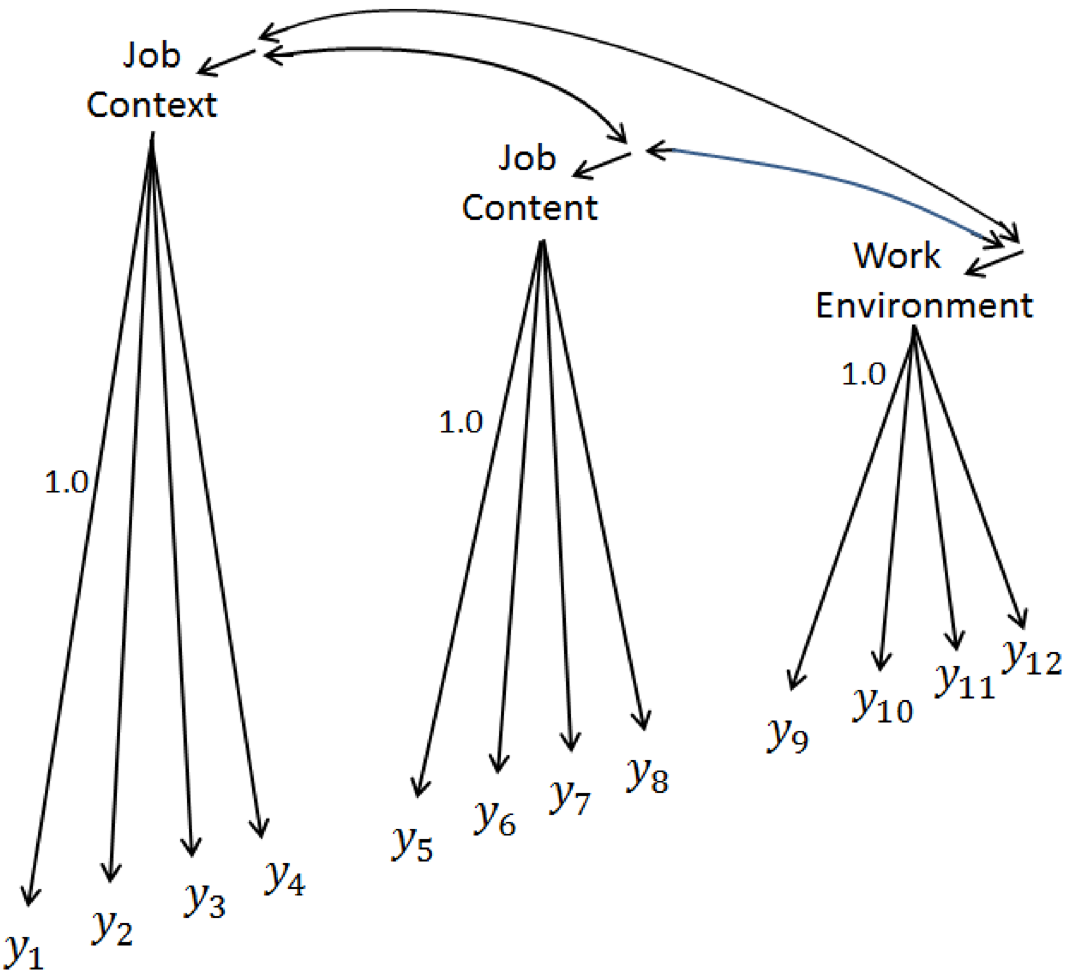

Figure 1: The Cheung and Lau (2012) configural model applies this model to each group.

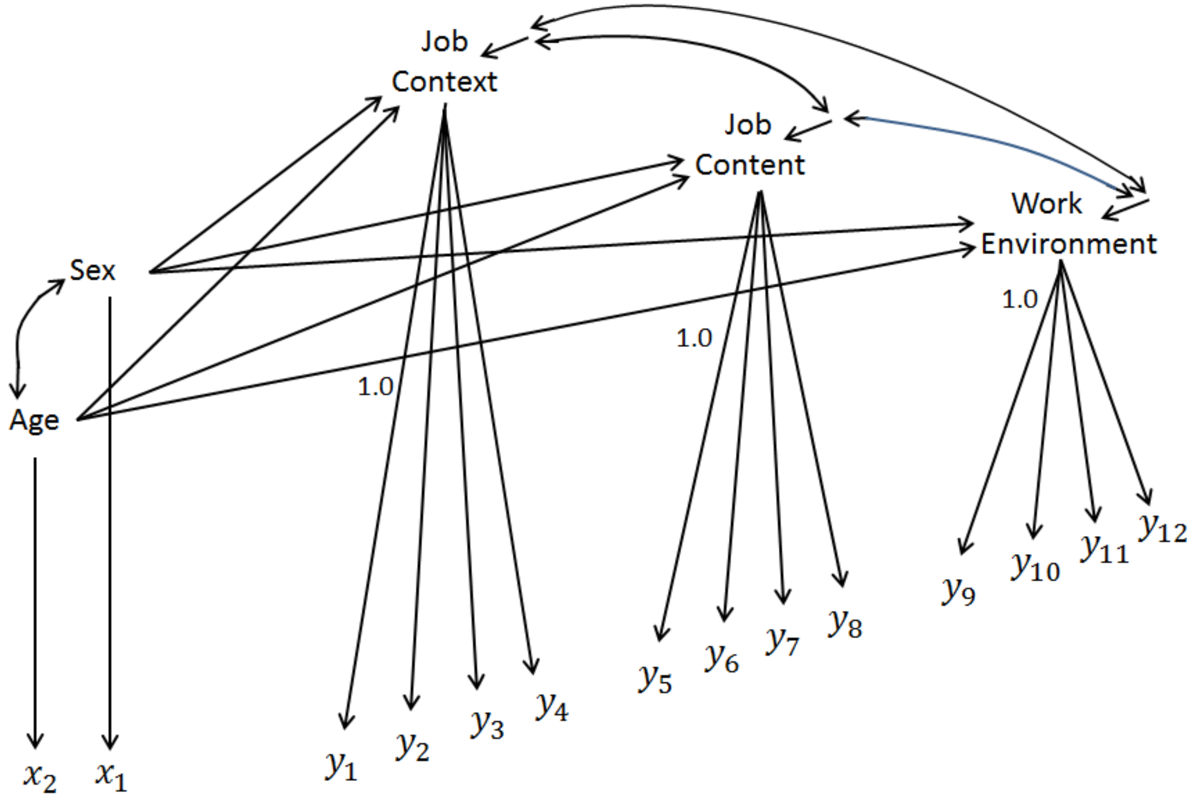

Figure 2: Sex and Age added to the Cheung and Lau (2012) configural model.

==

(***A post-review email exchange between Les Hayduk and James LeBreton***)

July 8, 2015

Dear Dr. LeBreton

I have now read your letter, Adam Meade's comments, and the reviewers' comments – several times. I submitted this manuscript to ORM because the problematic article I focused on appeared

in ORM. The reviewers' comments, and other context, made it abundantly clear that Adam Meade is correct when he says "the reviewers do not agree with the philosophy espoused in the manuscript". Unfortunately the philosophical disagreements seem to be over the same basic issue my manuscript reports as importantly problematic. My philosophy is that we must acknowledge and respond to evidence of inconsistency between our SE models -- including our "configural models" -- and the available data. (ORM's authors, reviewers, and editors seem to be into non-responding to evidence of model-data inconsistency in configural models.) I am unsure if you appreciate the depth or importance of this "philosophical" difference.

Given that you did not provide an opportunity to respond to the reviewers, or invite open debate by suggesting a target article with commentary and rejoinder, ORM currently seems unreachable from the "inside". Consequently, I am now planning to submit essentially this same manuscript to an open web journal (where the ORM reviewers' comments and possibly some ORM correspondence can be archived-attachments, and so future identified reviewers will have to make their comments in public) with specific reference to ORM and its entrenched problematic philosophy. I will try to make my ORM comments briefly, and that is likely to corner me into making some rather pointed statements, so I will have to document my "case" carefully. Consequently, I think I will have to refer to you as editor and Adam Meade as associate editor, on this manuscript -- the reviewers remain anonymous.

I would appreciate hearing any comments or suggestions you might wish to make.
Regards.

Les Hayduk

Les Hayduk; Department of Sociology; University of Alberta; Edmonton, Alberta;
Canada T6G 2H4   email: LHayduk@ualberta.ca   phone 780-492-2730

July 9, 2015

Hello Les,

I am sorry to hear that you were disappointed with the peer-review process and the outcome. Perhaps if I provided some additional context for the process and final decision, it might help alleviate some of your concerns.

First, I want to assure you that neither Cheung nor Lau were among the reviewers I asked to examine your paper. I wanted a fresh set of eyes to give an independent perspective on your manuscript.

Second, with that caveat in place, I can tell you that the manuscript was reviewed by some of the very best scholars in our field. These are individuals who are currently serving on the editorial boards of a number of leading journals in psychology, management, and quantitative methods. All three reviewers have served as chief editor or AE at one (or more) of the leading journals in

their fields. All three reviewers are acknowledged experts in measurement and applied psychology (i.e., all are elected fellows of APS and/or APA; with APA Fellowship occurring in Divisions 5 and/or 14). I could go on and on (distinguished career awards, citations, books, etc.); however, my basic point is that these individuals have extensive experience not only as authors and reviewers, but also as editors. Consequently, they are qualified to evaluate whether a paper appears to be on track for making an important methodological contribution to the organizational sciences (hence, my decision to solicit their input on your manuscript).

Third, normally we do not share the dimensional ratings provided by the reviewers; however, in the case of you paper, it might be helpful to summarize those evaluations. Perhaps this summary can provide some additional perspective on why a revision was not requested.

• On the dimension of "Appropriateness of Topic for Organizational Research Methods", the manuscript was evaluated as "Suitable" by two reviewers and "Highly Appropriate" by the third reviewer.
• On the dimension of "Conceptual Adequacy/Mastery of Relevant Literature", all three reviewers evaluated the manuscript as "Weak."
• On the dimension of "Technical Adequacy", all three reviewers evaluated the manuscript as having "Major Problems."
• On the dimension of "Clarity of Presentation", two reviewers evaluated the manuscript has having "Major Problems" and the third reviewer evaluated it as having "Minor Problems."
• On the dimension of "Significance of Contribution to the Field", the manuscript was evaluated as "Good" (which is roughly the midpoint of the rating scale) by one reviewer while the other reviewers evaluated the contribution as "None" and "Trivial", respectfully.
• Finally, when asked to judge the "Probability of Successful Revision" two reviewers suggested there was an only a 11-20% chance the paper could be successfully revised; the third reviewer was less optimistic and evaluated the paper has having a 0 to 10% chance.

Fourth, as editors, Adam and I look to the ratings of "significance/contribution" as the most important factor in determining whether to pursue a paper for revision or publication. As editors, it is our job to make the difficult (and at times unpleasant) decision concerning which papers appear to hold the greatest promise for achieving a successful revision, and which do not. The papers that are typically invited for revision are those evaluated as being on track for making clear and important contributions.

When deciding which papers to invite for revision, I try to respect the peer-review process. I try to identify the most qualified and talented individuals to review manuscripts and then carefully contemplate the counsel they have generously provided. I certainly understand your disappointment with the outcome, but this was not a situation where one reviewer misinterpreted some minor aspect of a paper and that minor misunderstanding resulted in the paper being rejected. This is a situation where all three reviewers and (the AE) agreed about the perceived limitations of a manuscript and the likelihood that those limitations could be adequately addressed in a revision.

You concluded your email by noting:

“I will try to make my ORM comments briefly, and that is likely to corner me into making some rather pointed statements, so I will have to document my "case" carefully. Consequently, I think I will have to refer to you as editor and Adam Meade as associate editor, on this manuscript -- the reviewers remain anonymous.”

Personally, I believe your time might be better spent trying to integrate the feedback you received from the reviewers into a repackaged/reframed manuscript (one that hopefully better highlights the contribution of your work) vs. ignoring/discounting that feedback and complaining about the process at another journal. In my own work, I have tried to follow the advice offered by John Hollenbeck (2008). Although I had not thought about the framing of manuscripts using the specific categories identified by John, I do believe he is 100% correct--consensus framing (either shifting or creating) typically yields a more compelling manuscript. This has been my experience as both an author and editor. So, you might ponder if there is a way to reframe your paper so that it better highlights the contribution of your work, perhaps using a consensus shifting or consensus creating frame.

• Hollenbeck, J. R. (2008). The role of editing in knowledge development: Consensus shifting and consensus creation. In Y. Baruch, A. M. Konrad, H. Aguinis, & W. H. Starbuck (Eds.), Opening the black box of editorship. New York: Palgrave MacMillan.

In any event, I am sorry that you were disappointed with the process and outcome at ORM. I hope that by sharing the above information, you now have a better sense of the factors that motivated our decision. I wish you the best of luck with this paper.

Sincerely,
James

--
James M. LeBreton
140 Moore Building
Department of Psychology
The Pennsylvania State University
University Park, PA 16802

E-mail: james.lebreton@psu.edu
Telephone: 814-865-9514
Facsimile: 814-863-7002
2015 CARMA Short Course Instructor (http://carma.wayne.edu)
Editor, Organizational Research Methods (http://orm.sagepub.com)
